# The loss of DNA polymerase epsilon accessory subunits POLE3–POLE4 leads to BRCA1-independent PARP inhibitor sensitivity

**DOI:** 10.1093/nar/gkae439

**Published:** 2024-06-03

**Authors:** Hasan Mamar, Roberta Fajka-Boja, Mónika Mórocz, Eva Pinto Jurado, Siham Zentout, Alexandra Mihuţ, Anna Georgina Kopasz, Mihály Mérey, Rebecca Smith, Abhishek Bharadwaj Sharma, Nicholas D Lakin, Andrew James Bowman, Lajos Haracska, Sébastien Huet, Gyula Timinszky

**Affiliations:** Laboratory of DNA Damage and Nuclear Dynamics, Institute of Genetics, HUN-REN Biological Research Centre, 6276 Szeged, Hungary; Doctoral School of Biology, University of Szeged, 6720 Szeged, Hungary; Laboratory of DNA Damage and Nuclear Dynamics, Institute of Genetics, HUN-REN Biological Research Centre, 6276 Szeged, Hungary; Department of Immunology, Albert Szent-Györgyi Medical School, Faculty of Science and Informatics, University of Szeged, 6720 Szeged, Hungary; HCEMM-BRC Mutagenesis and Carcinogenesis Research Group, Institute of Genetics, HUN-REN Biological Research Centre, 6276 Szeged, Hungary; Laboratory of DNA Damage and Nuclear Dynamics, Institute of Genetics, HUN-REN Biological Research Centre, 6276 Szeged, Hungary; Doctoral School of Multidisciplinary Medical Sciences, University of Szeged, Szeged, Hungary; Univ Rennes, CNRS, IGDR (Institut de génétique et développement de Rennes) - UMR 6290, BIOSITUMS 3480 Rennes, France; Univ Rennes, CNRS, IGDR (Institut de génétique et développement de Rennes) - UMR 6290, BIOSITUMS 3480 Rennes, France; Laboratory of DNA Damage and Nuclear Dynamics, Institute of Genetics, HUN-REN Biological Research Centre, 6276 Szeged, Hungary; Doctoral School of Multidisciplinary Medical Sciences, University of Szeged, Szeged, Hungary; Laboratory of DNA Damage and Nuclear Dynamics, Institute of Genetics, HUN-REN Biological Research Centre, 6276 Szeged, Hungary; Doctoral School of Multidisciplinary Medical Sciences, University of Szeged, Szeged, Hungary; Laboratory of DNA Damage and Nuclear Dynamics, Institute of Genetics, HUN-REN Biological Research Centre, 6276 Szeged, Hungary; Doctoral School of Multidisciplinary Medical Sciences, University of Szeged, Szeged, Hungary; Univ Rennes, CNRS, IGDR (Institut de génétique et développement de Rennes) - UMR 6290, BIOSITUMS 3480 Rennes, France; Department of Biochemistry, University of Oxford, South Parks Road, Oxford, UK; Department of Biochemistry, University of Oxford, South Parks Road, Oxford, UK; Division of Biomedical Sciences, Warwick Medical School, University of Warwick, UK; HCEMM-BRC Mutagenesis and Carcinogenesis Research Group, Institute of Genetics, HUN-REN Biological Research Centre, 6276 Szeged, Hungary; Univ Rennes, CNRS, IGDR (Institut de génétique et développement de Rennes) - UMR 6290, BIOSITUMS 3480 Rennes, France; Laboratory of DNA Damage and Nuclear Dynamics, Institute of Genetics, HUN-REN Biological Research Centre, 6276 Szeged, Hungary

## Abstract

The clinical success of PARP1/2 inhibitors (PARPi) prompts the expansion of their applicability beyond homologous recombination deficiency. Here, we demonstrate that the loss of the accessory subunits of DNA polymerase epsilon, POLE3 and POLE4, sensitizes cells to PARPi. We show that the sensitivity of POLE4 knockouts is not due to compromised response to DNA damage or homologous recombination deficiency. Instead, POLE4 loss affects replication speed leading to the accumulation of single-stranded DNA gaps behind replication forks upon PARPi treatment, due to impaired post-replicative repair. POLE4 knockouts elicit elevated replication stress signaling involving ATR and DNA-PK. We find POLE4 to act parallel to BRCA1 in inducing sensitivity to PARPi and counteracts acquired resistance associated with restoration of homologous recombination. Altogether, our findings establish POLE4 as a promising target to improve PARPi driven therapies and hamper acquired PARPi resistance.

## Graphical Abstract

**Graphical Abstract:**
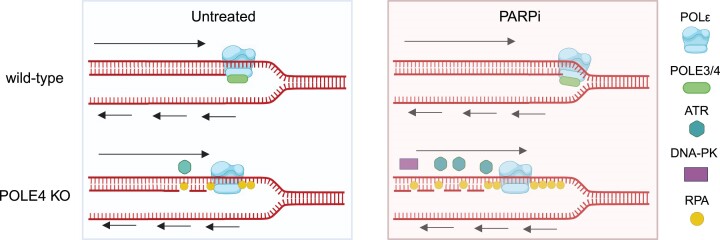

## Introduction

PARP inhibitors (PARPi) emerged as a promising therapeutic approach for the treatment of cancers with mutations in the breast cancer susceptibility genes BRCA1/2 nearly two decades ago (1,2). Breast cancer susceptibility protein 1 (BRCA1) and breast cancer susceptibility protein 2 (BRCA2) are pivotal for DNA double strand break (DSB) repair via the high-fidelity homologous recombination (HR) pathway. Mutations in the BRCA1/2 genes force the cells to rely on the error-prone non-homologous end joining (NHEJ) DSB repair, which leads to genomic instability (3).

PARP1 is the main writer of the posttranslational modification, ADP-ribosylation in response to DNA damage (4). PARP1 has a crucial role in the DNA damage response as it is recruited rapidly to the DNA lesions modifying itself and nearby targets by adding ADP-ribose moieties on specific protein residues. The poly (ADP-ribose) (PAR) chains generated by PARP1 trigger the recruitment of chromatin remodelers and DNA repair factors involved in early steps of the DNA damage response (5,6).

PARPi not only inhibit ADP-ribosylation signaling but also increase PARP1 retention on sites of DNA damage causing a so-called ‘PARP trapping’ phenomenon, which primarily underlies PARPi sensitivity (7). These PARP-bound DNA lesions are thought to be converted into DSBs during replication (8), leading to genomic instability and increased cell death in the case of HR deficiencies observed in cells displaying BRCA1/2 deficiency or a BRCAness phenotype (9). It is this Achilles’ heel that is exploited in the treatment of BRCA-deficient tumors with PARPi.

Since the approval of PARPi in the clinic, extensive work has been done to expand their therapeutic spectrum beyond the BRCAness phenotype. For example, synthetic lethality with PARPi has been reported upon loss of Histone PARylation Factor 1 (HPF1) (10), defects in the ribonucleotide excision repair pathway (11), impairments of resolving trapped PARP1 (12–15) and loss of factors of the Fanconi anemia pathway (16). More recently, PARPi sensitivity has been linked to the induction of single-stranded DNA (ssDNA) gaps either from unprocessed Okazaki fragments or unrestrained fork progression ultimately causing the cells to experience replication stress (17–19).

Unbiased knockout screens to identify genes underlying PARPi resistance suggested the loss of POLE3 and POLE4 to be synthetic lethal with PARPi (12,20,21). POLE3 and POLE4 are subunits of DNA polymerase epsilon (POLϵ). POLϵ is a protein complex mainly responsible for replicating the DNA leading strand during S phase (22). It consists of four subunits, the catalytic core composed of POLE1 along with POLE2 and the aforementioned accessory factors POLE3 and POLE4. Pol2 and Dpb2, the yeast orthologues of POLE1 and POLE2, respectively, are essential for viability but not Dpb3 (POLE4 in mammals) or Dpb4 (POLE3 in mammals) (23). Deletion of Dpb3 and Dpb4 does not stall replication but instead, reduces the processivity of the Pol2-Dpb2 subcomplex due to unstable binding to DNA leading to its frequent dissociation from the template which leaves gaps on the leading strand (24). This role in stabilizing the POLϵ complex becomes critical upon replication stress as shown by increased sensitivity to hydroxyurea (HU) upon loss of Dpb4 (25). In addition, while Dpb3 is important for normal cell-cycle progression (26), Dpb4 was reported to promote activation of the checkpoint kinase Mec1 (ATR in humans) upon replication stress (25). Importantly, similar sensitivity can be observed in mice fibroblasts lacking POLE4 (27).

**Figure 1.**
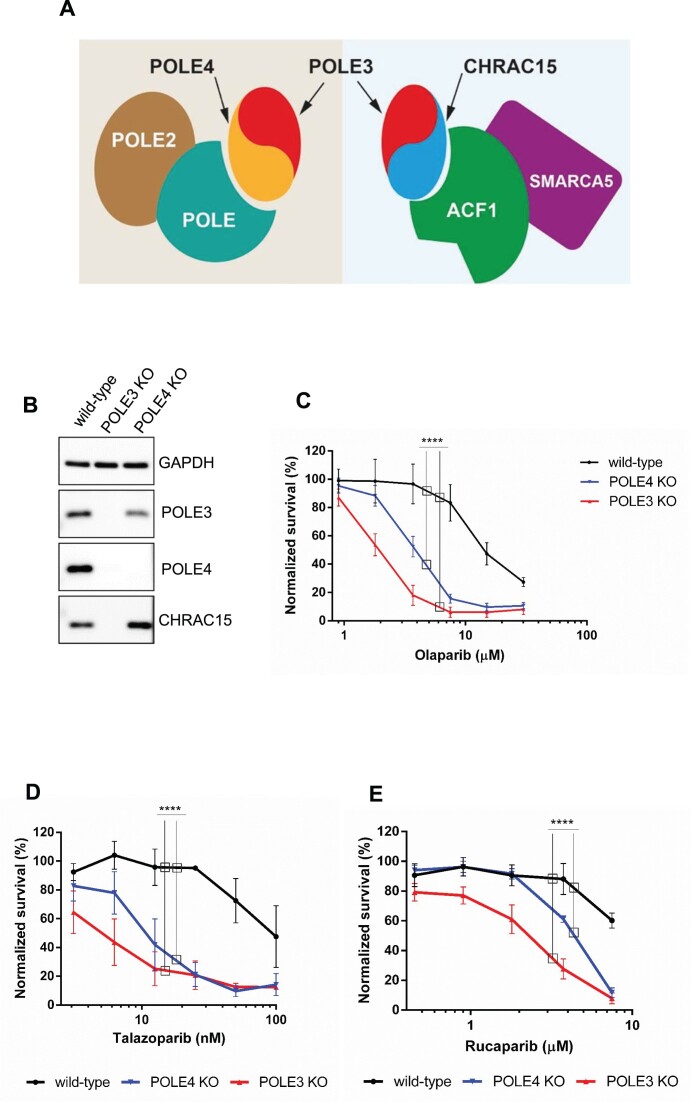
Loss of POLE3 or POLE4 induces PARPi sensitivity. (**A**) Schematic representation of the accessory subunits POLE3 and POLE4 within POLϵ and CHRAC complexes. (**B**) Western blot showing the levels of POLE3, POLE4 and CHRAC15 in HeLa wild-type, POLE4 KO and POLE3 KO cells. GAPDH is used as a loading control. (**C–E**) Cell survival assays demonstrating sensitivity of POLE3 KO and POLE4 KO to different PARPi compared to their parental HeLa wild-type. The curves are normalized to the untreated condition corresponding to each genotype. PARPi treatment was refreshed once during the 7-day long experiment. The graphs are derived from three independent experiments. Mean ± SEM (*n* = 3). Asterisks indicate *P*-values obtained by two-way ANOVA (**** *P*< 0.0001).

Both POLE3 and POLE4 have histone-fold domains and form a H2A–H2B-like heterodimer (28) which displays H3–H4 histone chaperone activity *in vitro* (29). More specifically, mice and yeast orthologs of POLE3 and POLE4 were shown to facilitate parental H3–H4 histone deposition on the leading strand keeping symmetrical segregation of histones between the two DNA strands (30,31). Consistent with their role in chromatin assembly, these accessory subunits were also shown to regulate heterochromatin silencing in budding and fission yeasts (32,33). Interestingly, the yeast ortholog of POLE3 (Dpb4) plays a dual role in this process depending on the complex it is part of (Figure 1A). On the one hand, as part of the POLϵ complex, the Dpb4-Dpb3 subcomplex ensures heterochromatin inheritance. On the other hand, within the yeast ortholog of the chromatin remodeling and chromatin-accessibility complex (CHRAC), the subcomplex Dpb4-Dls1 (CHRAC15 in humans) is important for the inheritance of an expressed state (32). As part of the CHRAC complex, Dpb4 also promotes histone removal at the vicinity of DSBs to facilitate DNA end resection (34), while through its interaction with Dpb3 in the POLϵ complex, it regulates the activation of the yeast checkpoint kinase Rad53 (CHK2 in humans), which is the effector kinase of Mec1/ATR in yeast (34,35).

PARP activity has been implicated in most of the POLϵ associated functions including DNA repair, replication, and chromatin regulation. In the present work, we provide insight into the mechanisms underlying the synthetic lethality observed upon loss of POLE4 and PARP inhibition.

## Materials and methods

### Cell lines and cell culture

Cell lines used in this study were cultured in DMEM (Biosera) supplemented with 10% FBS, 100 μg/ml penicillin, 100 U/ml streptomycin and 1% NEAA and maintained at 37°C in a 5% CO_2_ incubator unless otherwise stated. RPE-1 p53 KO and RPE-1 p53/BRCA1 double KO cells were kindly gifted from Alan D. D’Andrea lab (36) and were grown using DMEM-F12 (Biosera) supplemented with 10% FBS, 100 μg/ml penicillin, 100 U/ml streptomycin.

POLE3 KO and POLE4 KO cell lines were generated in this study from either wild-type HeLa cells or wild-type U2OS-FlpIn cells (kindly provided by Ivan Ahel's lab) using CRISPR/Cas9 technology. The HeLa cell line was authenticated by STR profiling (Eurofins Genomics) and had 100% match with HeLa (amelogenin + 12 loci) using the Cellosaurus cell line database (37).

The sgRNA sequences targeting either POLE3 or POLE4 are:

sgPOLE3: 5′-GTACAGCACGAAGACGCTGG-3′

sgPOLE4: 5′-GTCGGGATCTGCCTTCACCA-3′

### RNA interference and plasmid transfection

pSpCas9 (BB)-2A-Puro (PX459) V2.0 used to generate the knockouts of this study was a gift from Feng Zhang lab (Addgene, plasmid #62988) (38). Plasmid transfections were performed using Xfect (Takara) according to the manufacturer's protocol.

RNA interference experiments with siRNA (sequences in Supplementary Table S1) were conducted using Dharmafect (Dharmacon) or RNAiMAX (Lipofectamine) transfection reagents according to the manufacturers’ instructions. Downregulation was verified by western blotting using specific antibodies (detailed in Supplementary Table S2).

For rescue experiments the HeLa POLE4 KO cells were transfected with pmEGFP-C1 and either pcDNA5-FRT-TO or pcDNA5-FRT-TO-POLE4 using TransIT®-LT1 Transfection Reagent (Mirus) 24–48 h prior treatment. POLE4 expression was verified by Western blotting.

### PARP1 recruitment to sites of laser irradiation

HeLa wild-type or HeLa POLE4 KO cells were grown in 8-well Lab-Tek II chambered cover glass 30 (Thermo Scientific) and transfected 48 h prior to imaging with GFP-tagged PARP1 chromobody (Chromotek). For sensitization, growth medium was replaced with fresh medium containing 0.15 μg/ml Hoechst 33342 for 1 h at 37°C. Prior to imaging, the sensitizing medium was then replaced with CO_2_-independent imaging medium (Phenol Red-free Leibovitz's L-15 medium (Life Technologies) supplemented with 20% fetal bovine serum, 2 mM glutamine, 100 μg/ml penicillin and 100 U/ml streptomycin). For PARP inhibition conditions, cells were treated with Olaparib (30 nM) for 30 min prior to imaging.

Live-cell imaging experiments were performed on a Ti-E inverted microscope from Nikon equipped with a CSU-X1 spinning-disk head from Yokogawa, a Plan APO 60×/1.4 N.A. oil-immersion objective lens and a sCMOS ORCA Flash 4.0 camera. Laser microirradiation at 405 nm was done along a 16 μm-line through the nucleus using a single-point scanning head (iLas2 from Roper Scientific) coupled to the epifluorescence backboard of the microscope. The laser power at 405 nm was measured prior to each experiment to ensure consistency across the experiments and set to 125 μW at the sample level. Cells were kept at 37°C with a heating chamber. Protein recruitment was quantified using a custom-made Matlab (MathWorks) routine.

### Immunofluorescence

For native BrdU staining, cells were grown with 20 μM BrdU-containing medium for 48 h, the medium was then replaced with 10 μM Olaparib-containing medium for 24 h. Cells were washed with PBS, pre-extracted with 0.5% Triton X-100 in PBS for 5 min at 4°C then fixed with 4% paraformaldehyde (PFA) for 15 min at 4°C. Permeabilization was done using 0.5% Triton X-100 in PBS for 10 min followed by blocking with 5% FBS in 0.1% Triton X-100 for 45 min at room temperature, then incubated with primary antibody diluted in blocking solution overnight at 4°C.

For Rad51 experiments, cells were treated with 10 μM Olaparib-containing medium for the 48 h before being washed with PBS and pre-extracted with pre-extraction buffer (10 mM Tris–HCl, 2.5 mM MgCl_2_, 0.5% NP-40, 100× Protease inhibitor cocktail (Roche)) for 5 min at 4°C. Fixation was done using 4% PFA for 15 min at 4°C followed by permeabilization, blocking and antibody incubation as described earlier.

For S-phase PAR staining experiments, either wild-type or POLE4 KO cells were loaded with 2.5 μM of amine-reactive dye carboxyfluorescein diacetate, succinimidyl ester (CFSE) using the CellTrace™ CFSE Cell Proliferation Kit (Molecular Probes, Life Technologies) for 12 min at room temperature before seeding and mixing with the other unlabeled genotype. Cells were treated with DMSO (vehicle control) or PARGi (10 μM) or PARGi (10 μM) and Fen1i (10 μM) (Supplementary Table S3) for 1 h. Cells were pulse-labeled with the nucleotide analog EdU (10 μM) (5-ethynyl-2′-deoxyuridine, Baseclick, BCK-EdU555) for the last 20 min prior to fixing. Fixation and staining were done as described earlier.

Following overnight incubation with the primary antibodies (Supplementary Table S2), cells were washed three times with 0.1% Triton X-100 and incubated at room temperature with fluorescently tagged secondary antibody (Supplementary Table S2) for 1 h. Next, cells were washed with 0.1% Triton X-100 and counterstained with DAPI (1 μg/ml in PBS) for 10 min. To detect proliferating cells, EdU incorporation was visualized by a Click-IT Kit (Baseclick) according to the manufacturer's protocol.

Z-stacks of images were acquired on a Zeiss LSM800 confocal microscope with a Plan-Apochromat 20x/0.8 M27 or a water immersion Plan-Apochromat 40×/1.2 DIC M27 objective controlled by the ZEN 2.3 software. Fluorescence excitation was performed using diode lasers at 405, 488, 561 and 650 nm. Images were analyzed after generating maximum intensity projections of the z-stacks using a custom CellProfiler pipeline (39).

### BrdU comet post-replication repair assay

Exponentially growing cells were plated in 24-well plate at a density of 3 × 10^5^ cells/well. The following day the cells were pulse-labeled with 25 μM of the nucleotide analog BrdU, and incubated at 37°C for 30 min. Next, the cells were washed with PBS and treated or not with hydroxyurea (4 mM) for 3 h. Cells were then harvested (corresponding to 0 h repair) or left to perform post-replicative repair for additional 3 h. Cells were embedded in 0.75% low melting agarose and layered onto microscope slides (pre-coated with 1% agarose), covered with coverslips, and left to solidify for 5 min at 4°C.

The following steps were performed as detailed in (40) with slight modification detailed below. The alkaline lysis was performed in 0.3 M NaOH, 1 mM EDTA, pH 13 for 2 h in Coplin jars. The DNA was left to unwind for 40 min in this ice-cooled electrophoresis buffer. The electrophoresis was subsequently conducted at 1 V/cm (25 V, 300 mA) for 30 min in the same buffer at 10°C.

Following the electrophoresis, the slides were washed with neutralization buffer (0.4 M Tris–HCl, pH 7.4), blocked with PBS containing 1% BSA for 20 min at room temperature and incubated with the indicated primary antibody (Supplementary Table S2) for 2 h at room temperature. The primary antibody was washed off and slides were incubated with secondary antibody (Supplementary Table S2) for 2 h at room temperature then mounted by Fluoromount mounting solution containing DAPI, covered with coverslips and stored at 4°C until microscopy. Imaging was performed using Zeiss Axioscope Z2 fluorescent microscope. Scanning of images was done using automated scanning platform of Metasystem and the quantitation of comets was done by Metasystems Neon Metafer4 software. Three independent experiments were done with duplicate slides, 150–300 comet/slides were scored.

### BrdU comet assay for detection of ssDNA gaps

To detect the Olaparib-induced ssDNA gaps the comet method described above was applied with a slight modification. Exponentially growing cells were plated in 24-well plate at a density of 3 × 10^5^ cells/well in duplicates. The following day the growth medium was changed to fresh DMEM containing 25 μM of the nucleotide analog iododeoxyuridine (ldU), the cells were incubated at 37°C for 30 min. After the labelling, the cells were washed two times with PBS and were cultured in Olaparib (20 μM) containing medium for 24 h.

Cells were harvested by trypsinization, collected and pelleted in DMEM washed with ice-cold PBS. Pelleted again and resuspended in 500 μl ice cold H_2_O_2_ (75 μM, diluted in PBS), kept on ice for 3 minutes and pelleted by centrifugation (5 min, 900 rpm at 4°C). H_2_O_2_ was removed by washing twice with ice-cold PBS and, after the last centrifugation, cells were resuspended in 70 μl 0.75% low melting agarose/slide. The lysis and the further steps of the assay and analysis were identical to those detailed in the section of BrdU comet post-replication repair assay.

### DNA fiber assay

Exponentially growing cells were pulse labelled with 25 μM IdU for 20 min in 37C°, washed twice with prewarmed PBS and then labeled with 250 μM CldU (chlorodeoxyuridine) in the presence or absence of Olaparib (10 μM), cells were harvested, and DNA fiber spreads were prepared as described previously (41). Briefly, 2 μl of cells resuspended in PBS (10^6^ cells/ml) were spotted onto clean glass slides. Cells were lysed with lysis solution (0.5% sodium dodecyl sulphate (SDS) in 200 mM Tris–HCl (pH 7.5), 50 mM EDTA). Slides were tilted at 15° to the horizontal, allowing a stream of DNA to run slowly down the slide. Next, slides were air-dried for 20 minutes and fixed in methanol-acetic acid (3:1) and let dry for 10 min. Fixed fibers were rehydrated in water for 5 min and denatured (2.5 M HCl for 1 h) and blocked in blocking buffer (1% bovine serum albumin and 0.1% Tween20) for 1 h. Incubation with the two primary antibodies (Supplementary Table S2) was done for 2 h in humidified chamber at room temperature. Slides were washed and incubated with the secondary antibodies (Supplementary Table S2) for 90 min in humidified chamber at room temperature. DNA fibers were imaged using Axioscope Z2 fluorescent microscope (Zeiss, Germany) with a 60× objective. The lengths of DNA tracks corresponding to IdU and CldU labelling were measured using the Zen (Zeiss) software. In each experiment, a minimum of 200 independent fibers were analyzed per experiment. All measurements of four independent experiments were summarized in a dot plot created in GraphPad10.2.

### R-loop detection

Cells were treated with 10 μM Olaparib and 2 mM hydroxyurea for 24 h before being washed with PBS and fixed with MeOH for 15 min at −20°C. Cells were then treated or not with 2.5 units of RNase H (Thermo Fisher Scientific, cat #EN0201). Blocking was done with 3% BSA in 0.1% Triton-X100 for 45 min at room temperature, then cells were incubated with anti-DNA–RNA Hybrid primary antibody (Supplementary Table S2) diluted in blocking solution for overnight at 4°C. Next day, cells were washed three times with PBS and incubated at room temperature with anti-mouse IgG-Alexa Fluor 555 secondary antibody (Supplementary Table S2) for 1 h. Then the cells were washed with PBS and stained with DAPI (1 μg/ml in PBS) for 10 min. Z-stacks of images were acquired on a VisiScope Spinning Disk confocal microscope with 40×/0.6 objective using diode lasers at 405 and 561 nm. Detection was performed with Andor Zyla 4.2 PLUS camera. Fluorescent images were analyzed after generating maximum intensity projections of the z-stacks using CellProfiler (39).

### Cell survival assays

POLE4 KO, POLE3 KO and their parental wild-type cells were seeded in defined numbers in 96-well plates and treated for one week with Olaparib (0, 0.45, 0.9, 1.8, 3.7, 7.5, 15, 30 μM), Rucaparib (0, 0.45, 0.9, 1.8, 3.7, 7.5, 15 μM), Talazoparib (0, 15, 31, 62, 125, 250 nM) or ATRi (0, 0.6, 1.2, 2.5, 5, 10 μM) (Supplementary Table S3). For experiments with the combination of ATRi and Olaparib, 1 μM Olaparib was used along with 0.6 μM of ATRi. For experiments with RNAi-induced BRCA1 depletion the concentrations of Olaparib were 0, 0.3, 0.6, 1.2, 2.5, 5 and 10 μM. Treatment with HU (Sigma-Aldrich, 0, 0.5, 1, 2, 4, 8 mM) was for 24 h, with MMS (Sigma-Aldrich, 0, 0.0015, 0.003, 0.006, 0.012, 0.025, 0.05%) or with Etoposide (Sigma-Aldrich, 0, 0.45, 0.9, 1.8, 3.7, 7.5, 15 μM) for 1 h; following the indicated durations, cells were washed and incubated for 1 week in complete medium. After 7 days of incubation, the supernatants were aspirated and resazurin (Sigma) solution was added (25 μg/ml in Leibowitz's L-15, Gibco). The fluorescent resorufin product was measured after 30–60 min using a Biotek Synergy H1 microplate reader with a 530/590 filter set.

### Flow cytometry for intracellular markers and cell cycle analysis

Cells were dissociated with TrypLe Select (Gibco), washed with PBS and fixed with ice-cold ethanol. For labelling the intracellular markers, the cells were permeabilized and blocked with 0.5% Triton X-100 and 5% FBS in PBS, and then incubated with the appropriate primary antibody (Supplementary Table S2) overnight at 4°C. Next, the cells were washed two times with PBS, and incubated with fluorescently tagged secondary antibodies (Supplementary Table S2) for 2 h at room temperature. Finally, the DNA staining solution was added (10 μg/ml propidium-iodide and 10 μg/ml RNase in PBS) for 15 min at room temperature. The samples were analyzed with CytoFLEX S flow cytometer (Beckman Coulter Life Sciences) or FACSCalibur (Becton Dickinson). The measurements were evaluated with Kaluza Analysis software (Beckman Coulter Life Sciences).

### PARylation assay

Cells were cultured in 6 cm dishes. The cells were treated with H_2_O_2_ (2 mM) in fresh culturing medium for the indicated timepoints. At the time of collection, the cells were washed twice in PBS and lysed directly using denaturing lysis buffer (4% SDS, 50 mM Tris–HCl, pH 7.4, 100 mM NaCl, 4 mM MgCl_2_, 5 U/μl Benzonase). The cell lysates were collected using a cell scraper and the total protein concentration was equalized after measuring the initial concentration by NanoDrop (A280 setting). Samples were boiled in 4× Laemmli buffer for 5 min at 95°C prior to western blotting.

### Western blotting

Protein samples were prepared for SDS–polyacrylamide gel electrophoresis in 4× sample buffer (10% SDS, 300 mM Tris–HCl, 10 mM β-mercaptoethanol, 50% glycine, and 0.02% bromophenol blue). Separated proteins were blotted onto nitrocellulose or PVDF membranes, blocked for 1 h at RT in 5% low-fat milk or 5% BSA in 0.1% Tris-buffered saline, and incubated with primary antibodies overnight at 4°C. Horseradish peroxidase-conjugated secondary antibodies were used for 1 hour at room temperature. Membranes were developed with enhanced chemiluminescence using Odyssey Fc Imaging System (LI-COR Biotechnology).

### Statistical analysis

All experiments were done at least in triplicates and for immunofluorescence experiments at least 200 cells were scored. A minimum of 10 cells were irradiated in live-cell imaging experiments. Graphing and statistical analysis were done using GraphPad Prism versions 6 and 7. Statistical analysis of cell survival experiments was done using two-way ANOVA. PARP1 recruitment experiments were analyzed using Mann–Whitney unpaired *t*-test. Statistics for immunofluorescence experiments were performed using one-way ANOVA. Asterisks represent *P* values, which correspond to the significance (*P < 0.05, **P < 0.01, ***P < 0.001 and ****P < 0.0001).

## Results

### Loss of POLE3 or POLE4 causes PARPi sensitivity.

We and others have previously identified the loss of POLE3 and POLE4 to sensitize cells to the PARP1/2 inhibitor Olaparib (12,20,21). To confirm this finding, we employed CRISPR-Cas9 gene editing to generate POLE3 and POLE4 knock-outs (KO) in HeLa cells (Figure 1B, Supplementary Figure S1A). As expected, all tested clones of POLE3 KO and POLE4 KO were hypersensitive to Olaparib treatment in a cell survival assay (Figure 1C, Supplementary Figure S1B, C), a phenotype that was also observed upon knockdown of these subunits although to a lower extent (Supplementary Figure S1D). Furthermore, POLE3 and POLE4 KOs were also sensitive to other PARPi, such as Talazoparib and Rucaparib, showing that the sensitivity was not limited to Olaparib (Figure 1D, E). Additionally, the PARPi sensitivity upon POLE3 and POLE4 loss was not exclusive to HeLa cells as U2OS cells knocked out for POLE3 or POLE4 showed similar sensitive phenotype to Olaparib (Supplementary Figure S1E, F).

The heterodimerization of POLE3 and POLE4 is essential for their stability (21). Accordingly, the loss of either protein eliminated, or strongly reduced the levels of, the other (Figure 1A, B, Supplementary Figure S1A). POLE3 is a shared subunit between the POLϵ holoenzyme and the CHRAC complex (42), where POLE3 is forming heterodimer with POLE4 and CHRAC15, respectively (Figure 1A). Similar to the POLE3–POLE4 dimer, compromising the POLE3–CHRAC15 dimer by deleting POLE3 led to loss of CHRAC15 protein (Figure 1B). Since POLE3 KO cells lack both POLE4 and CHRAC15, we decided to further characterize the consequences of PARPi treatment only in the POLE4 KO to avoid confounding phenotypes arising from the lack of both POLE3–POLE4 and POLE3–CHRAC15 heterodimers.

### PARP1 is essential for Olaparib-induced POLE4 KO sensitivity with no apparent defects in the DNA damage response.

**Figure 2.**
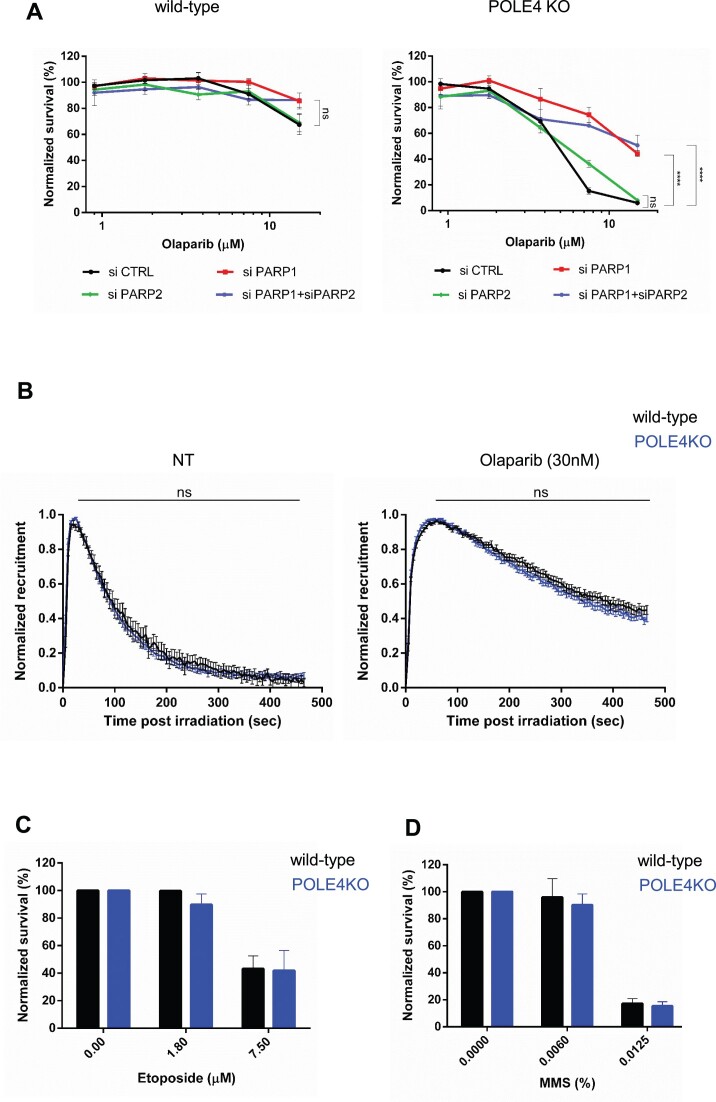
PARP1 is essential for Olaparib-induced POLE4 KO sensitivity with no apparent defects in the DNA damage response. (**A**) Cells survival assay showing Olaparib sensitivity of HeLa wild-type and POLE4 KO upon downregulation of PARP1, PARP2 or both of them using siRNA transfection. The curves are normalized to the untreated condition corresponding to each genotype. PARPi treatment was refreshed once during the 7-day long experiment. The graphs are derived from three independent experiments. Mean ± SEM (*n* = 3). Asterisks indicate *p-*values obtained by two-way ANOVA (ns. Not significant and **** *P*< 0.0001). (**B**) Normalized recruitment quantification of GFP-tagged PARP1 chromobody to sites of DNA damage in HeLa wild type and POLE4 KO cells in both untreated (left) or Olaparib treated (right) conditions. All data points included ± SEM. The figure is a representative experiment of three independent replicates (*n* = 3). Measurements were analyzed using Mann–Whitney unpaired *t*-test. (ns. Not significant). (C, D) Cell survival of HeLa wild-type and POLE4 KO cells upon treatment with etoposide (**C**) or MMS (**D**) for 1h. After the 1h treatment, cells were washed and incubated in culturing medium for 7 days. The bars are normalized to the untreated condition corresponding to each genotype. The graphs are derived from three independent experiments. Mean ± SEM (*n* = 3).

The toxicity of PARPi requires the presence of PARP1 in cells (7), with recent reports also highlighting a requirement of PARP2 (43,44). To investigate whether the sensitivity of POLE4 KO to PARPi was dependent on the presence of PARP1 or PARP2, we employed RNAi to deplete either or both factors. Cell survival assays demonstrated that the depletion of PARP1 alone was sufficient to rescue POLE4 KO sensitivity (Figure 2A, Supplementary Figure S2A). Instead, depleting PARP2 neither reduced the PARPi sensitivity nor further improved survival of the POLE4 KO co-depleted for PARP1, suggesting that the Olaparib-induced sensitivity of POLE4 KO relies on the presence of PARP1, but not PARP2, which is consistent with PARP1 trapping on their cognate lesions (Figure 2A, Supplementary Figure S2A).

Active ADP-ribosylation is crucial to release PARP1 from DNA. Cellular processes dampening or enhancing this signaling pathway lead to increased or reduced sensitivity to PARPi, respectively (45,46). Nevertheless, immunoblots showed no major difference in ADP-ribose (ADPr) levels between wild-type and POLE4 KO, as detected by a pan-ADPr reagent, both in the absence of genotoxic stress and after H_2_O_2_ treatment (Supplementary Figure S2B). This data indicates that POLE4 loss is not a source of DNA lesions that would lead to PARP1 activation. Moreover, it excludes POLE4 as playing a central role in the regulation of ADP-ribosylation signaling that could underlie the sensitivity of the KO cells to PARPi. Nevertheless, processes independent of ADP-ribosylation could also modulate PARP1 retention at DNA lesions (47,48). Thus, to more directly assess whether POLE4 regulates PARP1 mobilization from sites of DNA damage, we monitored the dynamics of endogenous PARP1 at sites of DNA damage upon laser micro-irradiation using a GFP-tagged PARP1-binding chromobody (49). In wild-type cells, PARP1 was recruited rapidly to sites of laser-induced damage before dissociating from the lesions within a time frame of a few hundreds of seconds (Figure 2B, Supplementary Figure S2C). As expected, this release was delayed upon Olaparib treatment (Figure 2B, Supplementary Figure S2C). PARP1 kinetics at sites of laser irradiation were similar in POLE4 KO and wild-type cells, irrespective of the presence of PARPi (Figure 2B, Supplementary Figure S2C) suggesting that POLE4 did not regulate PARP1 dynamics at sites of DNA damage.

To further investigate a potential role of POLE4 in DNA repair, we assessed the sensitivity of the POLE4 KO cells to genotoxic stress. POLE4 KO were not sensitive to methyl methanesulfonate (MMS) or etoposide treatments (Figure 2C, D). This is in line with earlier reports that observed no sensitivity to camptothecin (CPT) or MMS in a Dpb3-deficient yeast strain (50), or to ionizing radiation in POLE4-deficient mouse fibroblasts (27).

Altogether these data indicate that PARPi sensitivity of POLE4 KO is not a consequence of impaired PARP1 mobilization from sites of damage or defects in the DNA damage response.

### POLE4 loss alters the replication profile and increases the level of PARPi-induced ssDNA gaps

**Figure 3.**
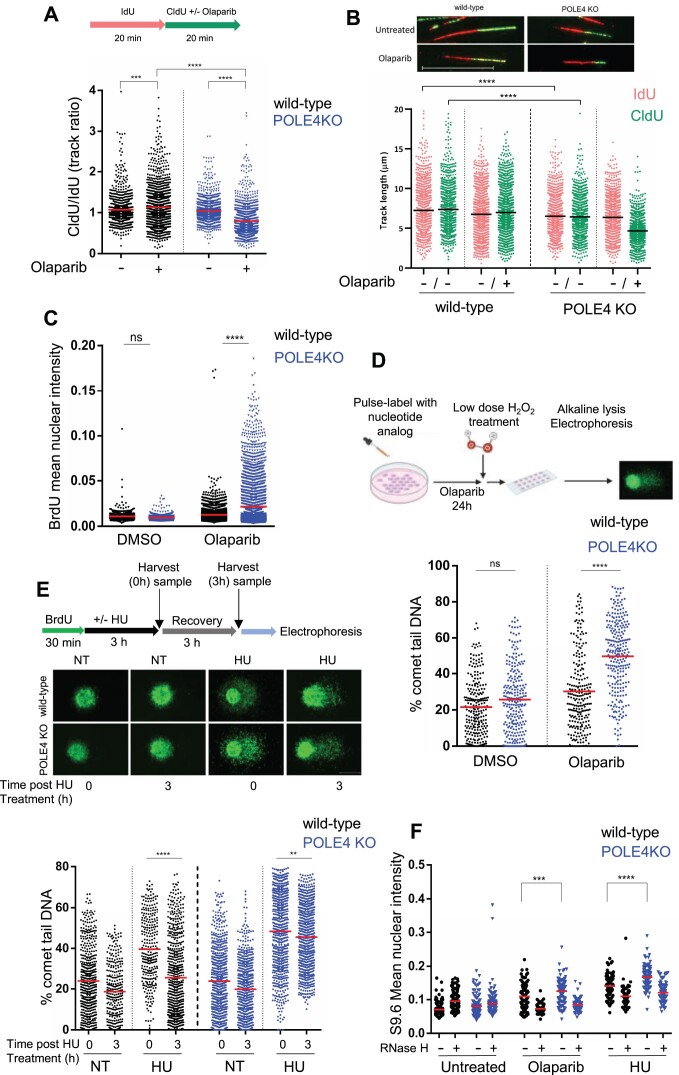
POLE4 loss alters the replication profile and increases PARPi-induced ssDNA gaps. (**A**, **B**) DNA fiber assay of HeLa wild-type and POLE4 KO cells upon treatment with Olaparib (20 μM). (A, top) Schematic illustration of the experimental procedure. (A, bottom) Results are illustrated as ratio of CldU and IdU labelled fiber track lengths obtained from four independent experiments (*n* = 4). (**B**, top) Representative images of fibers taken from HeLa wild-type and POLE4 KO cells with the indicated treatment. Scale bar, 20 μm. (**B**, bottom) Data represent length of the indicated tracks derived from four independent experiments (*n* = 4). Asterisks indicate *P*-values obtained by one-way ANOVA (*** *P*< 0.001, **** *P*< 0.0001). (**C**) Immunofluorescence experiment of native BrdU staining. Cells with the indicated genotypes were incubated with BrdU (20 μM, 48 h), then treated with Olaparib (10 μM, 24 h) or the control vehicle DMSO. Mean BrdU intensity of all scored cells was blotted. The graph represents one experiment out of three independent repetitions. Asterisks indicate *P*-values obtained by one-way ANOVA (ns, not significant, **** *P*< 0.0001). (**D**,top) Schematic illustration of BrdU-Comet experiment. (Bottom) Quantification of % of comet tail DNA of HeLa wild-type and POLE4 KO cells treated with Olaparib (20 μM, 24 h) or DMSO. The figure is a representative of three independent experiments. Asterisks indicate *P*-values obtained by one-way ANOVA (ns, not significant, **** *P*< 0.0001). (**E**, top) Schematic illustration of the post-replicative comet assay. Cells were BrdU pulse labelled, mock treated or treated with hydroxyurea (HU) (4 mM for 3 h) and harvested immediately (0 h) or left to recover for additional 3 h. Comet PRR assay and immunostaining was performed as detailed in Materials and methods. (Middle) Representative images of post-replication repair comet assay. Scale bar, 20 μm. (Bottom) Quantification of % of comet tail DNA. The figure is a representative of three independent experiments. Asterisks indicate *P*-values obtained by one-way ANOVA (** *P*< 0.01, **** *P*< 0.0001). (**F**) Detection of R-loops in untreated, Olaparib (10 μM) or hydroxyurea-treated (HU, 2 mM) wild-type or POLE4 KO HeLa cells after 24 h treatment. The graph shows the mean fluorescent intensity of nuclei with or without RNase H treatment. The figure is a representative of three independent experiments. Asterisks indicate *P*-values obtained by one-way ANOVA (*** *P*< 0.001, **** *P*< 0.0001).

PARPi have been reported to accelerate fork speed (51). Additionally, the accessory subunits of POLϵ were shown to be important for processive progression of the POLϵ complex on the DNA (24). To investigate the replication speed in POLE4 KO and how PARPi affects it, we employed DNA fiber assay, where cells were pulse labeled with the nucleotide analog IdU for 20 min followed by pulse labeling with another nucleotide analog, CldU for the same period of time with or without PARPi treatment (Figure 3A). As previously reported (51), PARPi treatment increased the fork speed in wild-type cells (Figure 3A) as shown by increased CldU/IdU ratios compared to untreated cells. Replication was slightly but significantly slower in POLE4 KO as compared to wild-type even without PARPi treatment (Figure 3B), indicated by the reduction in track lengths compared to wild-type cells, which can be attributed to impaired POLϵ progression as a consequence of POLE4 absence. Importantly, replication speed was further reduced in the presence of PARPi (Figure 3A, B), suggesting major defects in replication upon PARP inhibition.

Sensitivity to PARPi has been linked to defects in Okazaki fragments processing during DNA replication (17), and PAR signal was reported to correlate with the amount of unligated Okazaki fragments (52). To test whether POLE4 had a role in Okazaki fragments processing, we assessed PAR levels in replicating cells in the presence of Poly (ADP-ribosyl) glycohydrolase inhibitor (PARGi), which is required to detect the highly dynamic PAR signal at replication foci (52). To ensure each cell line undergoes the exact same conditions when performing the experiment and imaging, we loaded POLE4 KO cells with the amine-reactive carboxyfluorescein diacetate, succinimidyl ester (CFSE) cell tracker dye and mixed it with its wild-type unlabeled cells. The lack of difference in PAR levels between POLE4 KO and wild-type cells suggested similar amounts of Okazaki fragments in both cell lines (Supplementary Figure S3A). Co-inhibition of PARG and Fen1, an enzyme responsible for processing Okazaki fragments (52), increased further the PAR signal compared to PARG inhibition alone but again to comparable levels in both wild-type and POLE4 KO (Supplementary Figure S3A), indicating that POLE4 loss neither altered the processing of Okazaki fragments nor led to increased S-phase specific PARP activity.

PARPi were reported to induce ssDNA gaps behind replication forks through the suppression of fork reversal (18). To test whether such gaps were formed upon Olaparib treatment in cells lacking POLE4, we employed the non-denaturing BrdU immunostaining assay, where the specific antibody against BrdU is unable to bind the nucleotide analog in native conditions unless there is a single stranded DNA gap opposite it, therefore making the intensity of BrdU staining an indicator of the ssDNA gaps levels in the cell (17,18). With this assay, POLE4 KO cells displayed a striking increase in the intensity of BrdU staining upon Olaparib treatment compared to their wild-type counterparts, indicating a dramatic accumulation of unprocessed ssDNA gaps in these cells (Figure 3C, Supplementary Figure S3B). To further confirm this observation, we performed a variation of a comet assay, the so called BrdU comet assay, where pulse labeling cells with a nucleotide analog is used to highlight newly synthesized DNA, rendering the assay S-phase specific (40,53). The assay was further modified by application of a short, low dose of H_2_O_2_ treatment immediately prior to embedding the cells in agarose, to convert single stranded gaps into double stranded breaks, the resulting fragmented DNA fraction thus migrating into the comet tail (53). Consistent with the non-denaturing BrdU staining results (Figure 3C, Supplementary Figure S3B), we observed a significant increase in the percentage of comet tail DNA of POLE4 KO cells treated with Olaparib, revealing increased formation of ssDNA gaps (Figure 3D, Supplementary Figure S3C), indicating the role of POLE4 in averting the formation of PARPi-induced ssDNA gaps.

The accumulation of ssDNA gaps could also reflect impaired post-replicative repair (PRR). To test whether POLE4 plays a role in this process, we performed a comet assay variant described previously as a sensitive indicator of PRR efficiency (40). Briefly, cells were pulse-labeled with BrdU and subjected to Hydroxyurea (HU) treatment to induce S-phase specific ssDNA breaks, then the treatment was washed out and cells were incubated for a period of time to allow the repair of replicative gaps. In wild-type cells, HU treatment led to a higher level of post-replicative gaps as compared to their untreated counterparts, indicated by an increase of percentage of DNA in the comet tails, that was reduced after the 3h recovery time (Figure 3E). On the contrary, POLE4 KO cells showed increased levels of post-replicative gaps upon HU treatment, even after 3h recovery as compared to wild-type cells (Figure 3E), underscoring the role of POLE4 in maintaining efficient PRR. Accordingly, POLE4 KO were hypersensitive to replication stress induced by HU treatment in cell survival assays (Supplementary Figure S3D), in line with previous reports (21,54,55). It has been reported that HU treatment leads to the accumulation of R-loops (56), DNA:RNA hybrids that form due to nascent RNA binding to its complementary DNA, reflecting a possible replication-transcription conflict (57). Moreover, PARP1 was shown to be important for R-loops resolution (58–60). Given POLE4 KO sensitivity to both PARPi and HU, we probed for the levels of R-loops in these cells using the S9.6 antibody that recognizes DNA:RNA hybrids (61). Loss of POLE4 caused increased accumulation of R-loop structures compared to wild-type cells upon treatment with either HU or PARPi (Figure 3F, Supplementary Figure S3E) indicating a role of POLE4 in resolving these structures. The pan nuclear intensity observed with this antibody decreased upon treatment with RNase H, emphasizing its specificity for the R-loops (Figure 3F, Supplementary Figure S3E).

Taken together, these results demonstrate that the loss of POLE4 alters replication profile by slowing down nucleotide incorporation, a phenotype that gets exacerbated upon PARPi, leading to accumulation of ssDNA gaps due to impaired processing of post-replicative gaps and defects in resolving of R-loops.

### POLE4 protects against replication stress induced by PARPi and ATRi

**Figure 4.**
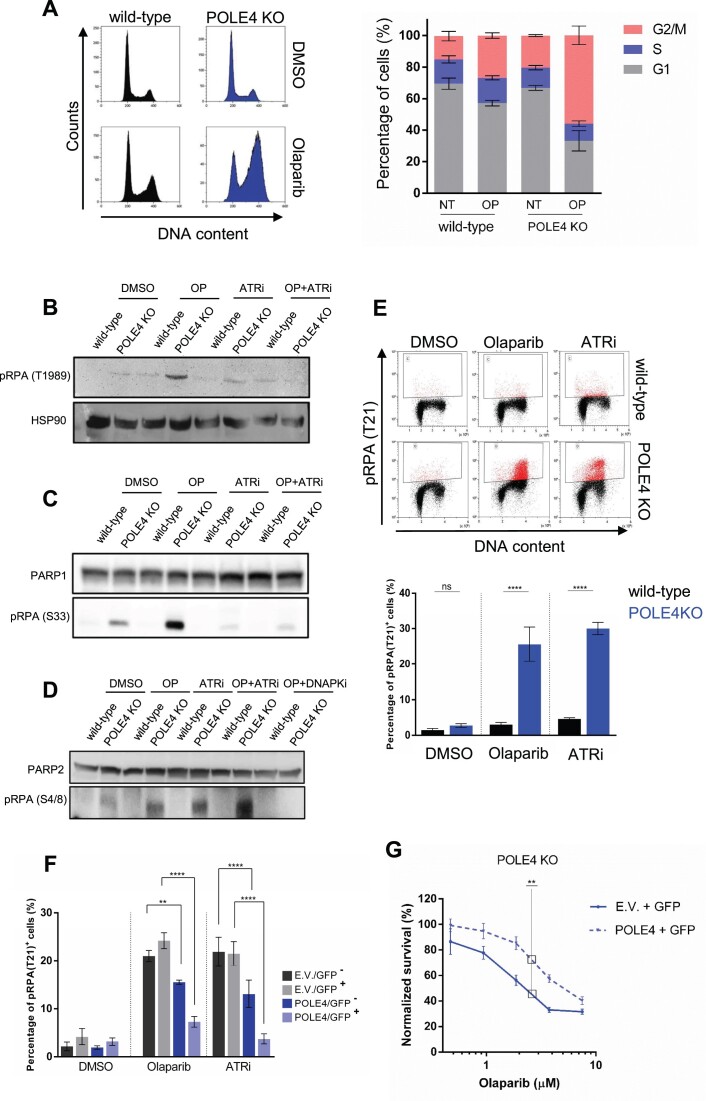
POLE4 protects against replication stress induced by PARPi and ATRi. (**A**, left) Representative FACS experiment showing cell-cycle profile of cells with the indicated genotypes with or without Olaparib treatment (5 μM, 24 h). (Right) Percentages of cells in G1, S or G2/M cell cycle phases are presented as mean ± SEM of three independent experiments (*n* = 3). (**B**, **C**) A representative western blot out of three independent repetitions of HeLa wild-type and POLE4 KO cells with the indicated antibodies upon treatment with Olaparib (5 μM, 24 h), ATRi (5 μM, 24 h) or both of them. HSP70 and PARP1 are used as a loading control. (**D**) A representative western blot out of three independent repetitions of HeLa wild-type and POLE4 KO cells with the anti-pRPA (S4/8) upon treatment with Olaparib (5 μM, 24 h), ATRi (5 μM, 24 h), their combination or Olaparib plus DNA-PKi (5 μM, 24 h). PARP2 is used as a loading control. (**E**) Flow cytometry of HeLa wild-type or POLE4 KO cells after culturing for 24 h with Olaparib (5 μM), ATRi (5 μM) or DMSO. The cells were fixed and stained with anti-pRPA (T21) and propidium-iodide (DNA content). (Top) FACS images of a representative experiment are shown. (Bottom) Bar chart shows the mean ± SEM of percentages of pRPA(T21) positive cells from three independent experiments (*n* = 3). Asterisks indicate *P*-values obtained by one-way ANOVA (ns, not significant, **** *P*< 0.0001). (**F**) Percentage of pRPA (T21) positive cells upon transient POLE4 expression. POLE4 KO cells were transfected with GFP and empty (E.V.) or POLE4 coding plasmids for 48 h, then treated with Olaparib (5 μM), ATRi (5 μM) or with DMSO for 16 h. The cells were fixed and stained with anti-pRPA (T21) and propidium-iodide. RPA (T21) phosphorylation is shown in cells gated to GFP positive and negative populations during flow cytometry analysis. Data are mean ± SEM from three independent experiments (*n* = 3). Asterisks indicate *P-*values obtained by one-way ANOVA (** *P*< 0.01, **** *P*< 0.0001). (**G**) Cell survival assay showing Olaparib sensitivity of POLE4 KO cells transfected with GFP and empty (E.V.) or POLE4 coding plasmids. Olaparib treatment was refreshed once during the 6-day long experiment. The graphs show the relative survival normalized to the untreated samples of each transfection. Data are mean ± SEM (*n* = 3) of triplicate samples from one representative out of three independent experiments. Asterisks indicate *P-*values obtained by two-way ANOVA (** *P*< 0.01).

Accumulation of unprocessed ssDNA gaps induces replication stress and alters cell cycle progression (62). To assess whether POLE4 KO cells stall at a certain phase of the cell cycle, we probed their cell cycle profile. All POLE4 KO clones showed mild accumulation in G2/M without treatment, which was strongly elevated upon PARPi treatment (Figure 4A, Supplementary Figure S4A), suggesting already a low level of replication stress in the absence of POLE4, which gets strongly potentiated upon PARP inhibition. During DNA replication, the phosphatidylinositol 3-kinase-related kinase (PIKK) family member Ataxia-telangiectasia mutated and RAD3-related (ATR) orchestrates origin firing, protects replication forks and regulates cell cycle progression (63). Indeed, we detected that ATR activity was mildly increased in POLE4 KO cells as compared to wild-type, revealed by autophosphorylation of residue Threonine 1989 (T1989), a proxy for ATR activation (64) (Figure 4B). PARPi treatment strongly enhanced the pATR signal, which was reversed by ATR inhibitor (ATRi), indicating a specific role of ATR in mediating PARPi-induced replication stress response (Figure 4B).

Our results revealed that the replication profile of POLE4 KO is impaired, which is potentiated by PARPi treatment leading to the accumulation of ssDNA gaps and replication stress induction. Replication protein A (RPA) acts as a sensor of ssDNA and signals different levels of replication stress by getting phosphorylated sequentially on multiple residues by PIKKs, with Serine 33 (S33) being phosphorylated early on upon mild replication stress, followed by Serine 4 and 8 (S4/8) and Threonine 21 (T21) upon severe replication stress (65). Consistent with mild replication stress upon the loss of POLE4, we detected pRPA (S33) signal in untreated POLE4 KO cells, which got exacerbated upon PARPi (Figure 4C). The pRPA (S33) signal was abolished by ATRi, in line with being phosphorylated by ATR (65). Furthermore, we detected pRPA (S4/8) signal in POLE4 KO cells without any treatment, which was enhanced upon PARPi, ATRi and their combination (Figure 4D) indicating elevated levels of replication stress. This signal was suppressed by treatment with DNA-dependent protein kinase (DNA-PK) inhibitor (DNA-PKi) (Figure 4D), consistent with the phosphorylation of this residue by DNA-PK (65). ATRi-induced severe replication stress in POLE4 KO is also reflected in their reduced survival (Supplementary Figure S4B), indicating a synthetic lethal interaction between ATR inhibition and POLE4 deficiency in line with previous reports (21,55).

The inability of POLE4 KO to resolve replication stress led to increased severity of the phenotype as PARPi treatment caused a strong increase in the pRPA (T21) signal, which was characteristic to the G2/M arrested population in POLE4 KO, but not detected in wild-type cells (Figure 4E). This phenotype was also observed in all other POLE4 KO clones (Supplementary Figure S4C), confirming that it was not clone-dependent. Interestingly, RPA phosphorylation on T21 residue was also induced by ATR inhibition (Figure 4E), suggesting a severe replication stress, which may ultimately lead to replication catastrophe due to accumulation of DSB indicated by phosphorylation of serine 139 on the histone variant H2AX (also called γH2A.X). We measured γH2A.X level upon MMS-induced DSB as a positive control. As expected, MMS treatment induced γH2A.X signal, however, there was no significant difference in the percentage of γH2A.X positive cells between wild-type and POLE4 KO cells (Supplementary Figure S4D), which is in line with our previous observation that POLE4 KO and wild-type cells were equally sensitive to MMS in a cell survival assay (Figure 2D). While PARPi treatment did not lead to DSB in wild-type or in POLE4 KO as indicated by low levels of γH2A.X (Supplementary Figure S4D), ATRi caused a dramatic increase in the percentage of γH2A.X positive cells only in POLE4 KO (Supplementary Figure S4D) revealing differences in the cellular response to PARPi and ATRi. It has been reported that ATRi can induce ssDNA accumulation, which can be converted to DSB leading to phosphorylation and activation of other PIKKs, such as DNA-PK and Ataxia-telangiectasia mutated (ATM) (63).

To address the role of POLE4 in protecting against severe replication stress, we measured the pRPA (T21) signal in POLE4 KO cells co-transfected with a plasmid expressing untagged POLE4 or an empty vector together with a GFP-expressing plasmid and subjected these cells to treatment with either PARPi or ATRi. After transfection, we sorted the cells using FACS into two populations: one that was GFP^+^, likely to have POLE4 expression as well, and one that was GFP^−^ which corresponded to cells that were likely not expressing POLE4 (Supplementary Figure S4E, F). In samples co-transfected with the GFP expressing plasmid and empty vector, the pRPA (T21) signal was detected in POLE4 KO upon either PARPi or ATRi treatment in both GFP positive and negative populations (Figure 4F), indicating that GFP expression itself does not alter the level of replication stress in POLE4 KO. In cells co-transfected with the plasmids expressing GFP and POLE4, pRPA (T21) was detected upon treatment with either PARPi or ATRi in the GFP^−^ population corresponding to no POLE4 expression. Importantly, this phenotype was rescued in the GFP^+^ population, where POLE4 was expressed (Figure 4F, Supplementary Figure S4E, F). The transient expression of POLE4 in POLE4 KO also reduced their sensitivity to PARPi as compared to the empty vector control (Figure 4G).

Altogether, these results demonstrate that POLE4 ensures normal cell-cycle progression and curbs replication stress associated with PARPi or ATRi treatment.

### Distinct roles of PIKKs in response to PARPi-induced replication stress in POLE4 KO

**Figure 5.**
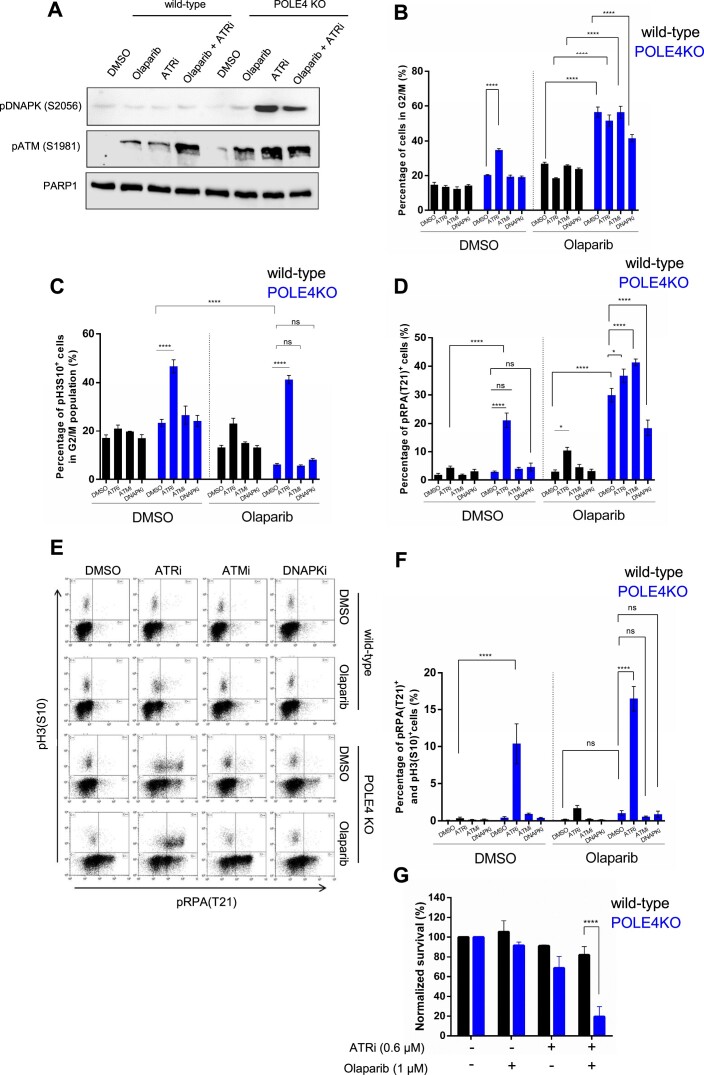
Distinct roles of PIKKs in response to PARPi-induced replication stress in POLE4 KO. (**A**) A representative Western blot out of three independent repetitions of HeLa wild-type and POLE4 KO cells with the indicated antibodies upon treatment with Olaparib (5 μM, 24 h), ATRi (5 μM, 24 h) or both of them. PARP1 is used as a loading control. (**B**) Percentages of HeLa wild-type and POLE4 KO in G2/M phase after 24 h of Olaparib (5 μM) and/or ATRi (5 μM), ATMi (5 μM) DNA-PKi (5 μM) treatment. DMSO was used as a solvent control. Percentages of cells in G2/M were determined from cell cycle analysis measured by flow cytometry. Bar chart shows the mean ± SEM from five independent experiments (*n* = 5). Asterisks indicate *P*-values obtained by one-way ANOVA (**** *P*< 0.0001). (**C**) Percentages of HeLa wild-type and POLE4 KO in mitosis after 24h treatment with Olaparib (5 μM) and/or ATRi (5 μM), ATMi (5 μM), DNA-PKi (5 μM). DMSO was used as a solvent control. The cells were stained with anti-pH3(S10) and propidium-iodide, and the ratio of mitotic cells was determined by pH3(S10) positivity within the G2/M gate in flow cytometry. Bar chart shows the mean ± SEM from three independent experiments (*n* = 3). Asterisks indicate *P*-values obtained by one-way ANOVA (ns, not significant, **** *P*< 0.0001). (**D**) Percentages of phospho-RPA (T21) positive HeLa wild-type or POLE4 KO cells after 24 h of Olaparib (5 μM) and/or ATRi (5 μM), ATMi (5 μM), DNA-PKi (5 μM) treatment. DMSO was used as a solvent control. The cells were stained with anti-pRPA (T21) and propidium-iodide (DNA content), and the percentages of pRPA (T21) positive cells were determined by flow cytometry. Bar chart shows the mean ± SEM of percentages of pRPA (T21) positive cells from four independent experiments (*n* = 4). Asterisks indicate *P*-values obtained by one-way ANOVA (ns, not significant, * *P*< 0.05, **** *P*< 0.0001). (**E**, **F**) Mitotic entry of pRPA (T21) positive HeLa wild-type or POLE4 KO cells after 24 h of Olaparib (5 μM) and/or ATRi (5 μM), ATMi (5 μM), DNA-PKi (5 μM) treatment. DMSO was used as a solvent control. The cells were stained with anti-pH3(S10) and anti-pRPA (T21) and analyzed by flow cytometry. (**E**) FACS image of a representative experiment is shown. (**F**) Bar chart shows the mean ± SEM of percentages of pRPA (T21)/pH3(S10) double positive cells from three independent experiments (*n* = 3). Asterisks indicate *P*-values obtained by one-way ANOVA (ns, not significant, **** *P*< 0.0001). (**G**) Cell survival assay of HeLa wild-type and POLE4 KO cells treated with Olaparib (1 μM) and/or ATRi (0.6 μM). The columns represent normalized survival of the cells upon the indicated treatments. The treatment was refreshed once during the 7-day experiment. Mean ± SEM (*n* = 3). The figure is derived from three independent experiments. Asterisks indicate *P-*values obtained by two-way ANOVA (**** *P*< 0.0001).

Our results suggest the role of other members of the PIKK family besides ATR in replication stress signaling in the POLE4 KO. Therefore, we probed for the activity of ATM and DNA-PK as well. While DNA-PK activity, as revealed by its autophosphorylation, was not changed in wild-type cells upon Olaparib and/or ATRi treatment, it was markedly elevated in POLE4 KO after 24 h of ATRi treatment alone or when combined with Olaparib (Figure 5A). ATM phosphorylation (pATM)—an indicator of ATM activation—was modestly elevated upon incubation of the wild-type cells either with Olaparib or with ATRi alone, while strongly enhanced in the presence of their combination. Compared to wild-type cells, the ATM phosphorylation was further increased in POLE4 KO cells both in the case of single Olaparib or ATRi treatment and when combined (Figure 5A). These results reveal the complex interplay between PIKKs in POLE4 KO cells in response to replication stress.

Next, we aimed to reveal the role of PIKKs in the PARPi-induced cell cycle arrest. The cell cycle of wild-type cells was not affected by either ATRi, ATMi or DNA-PKi alone, and Olaparib treatment caused a mild G2/M accumulation with or without PIKK inhibitors (Figure 5B, Supplementary Figure S5A). In line with the ATRi sensitivity of POLE4 KO, ATRi treatment alone, but not the other PIKK inhibitors, caused a marked accumulation of G2/M cells in POLE4 KO (Figure 5B, Supplementary Figure S5A). In contrast to ATR and ATM inhibition that had little effect on Olaparib-induced cell cycle arrest in POLE4 KO, DNA-PKi reduced it (Figure 5B, Supplementary Figure S5A) indicating a role of DNA-PK activation in the cell-cycle arrest upon PARPi in POLE4 KO.

To refine whether Olaparib leads to G2 or mitotic arrest, we quantified a mitotic marker, histone H3 phosphorylated on Serine 10 (pH3S10). Despite the strong accumulation of cells with DNA content characteristic to G2/M detected in Olaparib-treated POLE4 KO, the ratio of mitotic cells within the G2/M population was reduced (Figure 5C, Supplementary Figure S5B). This shows that POLE4 KO cells are arrested in G2 upon PARP inhibition, presumably due to the accumulation of ssDNA gaps and ATR activation. ATR inhibition promoted the transition of POLE4 KO cells into mitosis, even if PARP1 was inhibited (Figure 5C, Supplementary Figure S5B) confirming that ATR is the major checkpoint kinase responsible for the G2/M cell cycle arrest in POLE4 KO. The proportion of mitotic cells did not change in the presence of other PIKK inhibitors (Figure 5C, Supplementary Figure S5B).

As pRPA (T21) is a marker of severe replication stress and it overlapped with G2/M arrest in PARPi-treated POLE4 KO cells, we aimed to address which PIKK was responsible for this signal. Of the tested PIKK inhibitors, only ATRi increased the percentage of pRPA (T21) positive wild-type cells upon Olaparib treatment significantly (Figure 5D, Supplementary Figure S5C). As we have shown previously, single ATRi treatment induced pRPA (T21) phosphorylation (Figure 4E), but neither ATMi nor DNA-PKi treatment alone changed the fraction of pRPA (T21) positive POLE4 KO cells (Figure 5D, Supplementary Figure S5C). On the other hand, all PIKK inhibitors altered the percentage of pRPA (T21) positive POLE4 KO to Olaparib: ATRi and ATMi increased the percentage of pRPA positive cells, while DNA-PKi decreased it (Figure 5D, Supplementary Figure S5C). The double staining of pH3 (S10) and pRPA (T21) also revealed that the Olaparib-induced pRPA (T21) positive POLE4 KO cells were blocked in G2 and they were prevented from entering mitosis, unless released from the control of ATR (Figure 5E, F). ATR inhibition allowed the cells with persisting replication defects to prematurely enter mitosis leading to replication catastrophe as revealed by γH2A.X positive cells (Supplementary Figure S4D) and reduced cell survival (Supplementary Figure S4B) and (54,66). In support of this, while the treatment with low dose of either Olaparib or ATRi alone had minor effect on the survival of POLE4 KO cells, combining them synergistically killed POLE4 KO cells (Figure 5G). This establishes the loss of POLE4 as a major sensitizing event to co-treatment with ATRi and PARPi, a drug combination being tested in clinical trials (67).

Together, these results emphasize the importance of ATR signaling in restraining POLE4 KO from entering mitosis upon PARPi-induced replication stress. Furthermore, our data reveal that DNA-PK activity contributes to the replication stress observed in POLE4-deficient cells.

### POLE4 acts parallel to BRCA1 in inducing PARPi sensitivity

**Figure 6.**
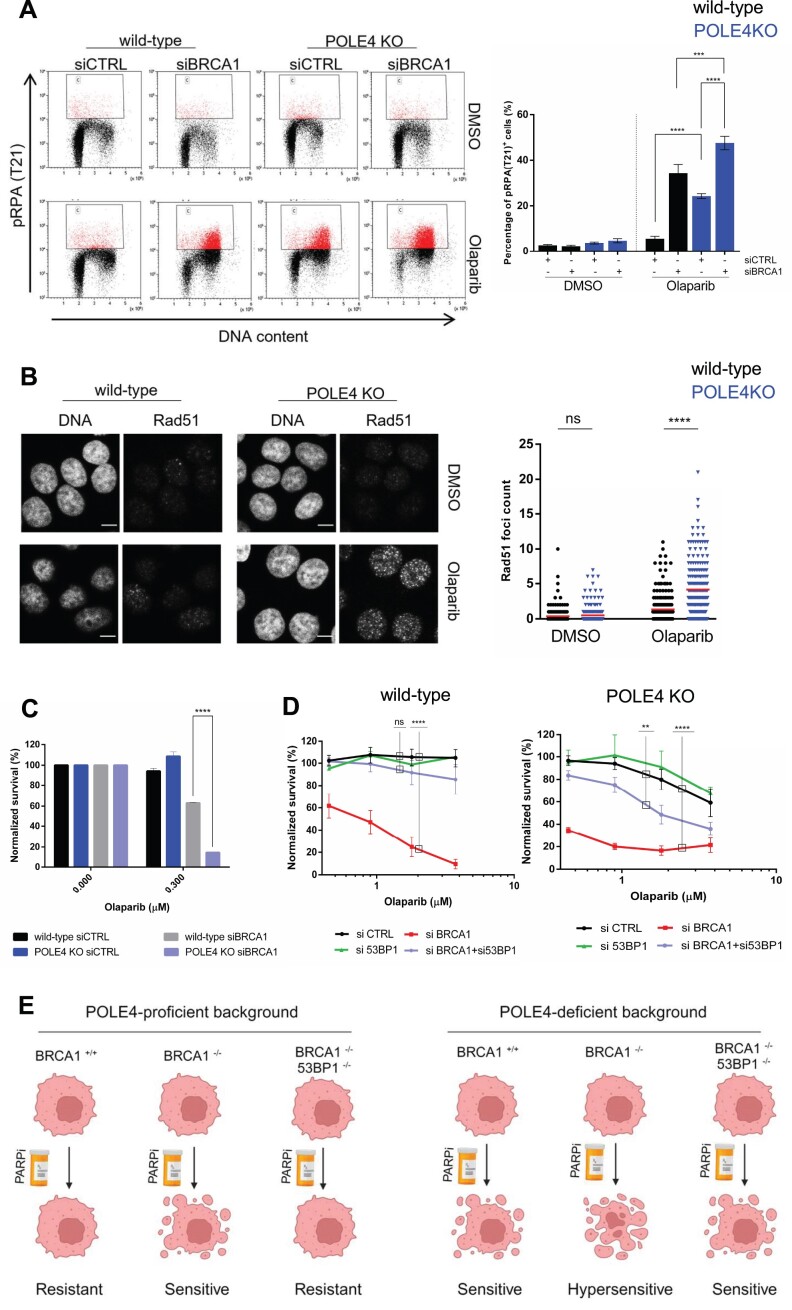
POLE4 acts parallel to BRCA1 in inducing PARP inhibitor sensitivity. (**A**) Flow cytometry of HeLa wild-type or POLE4 KO cells with downregulated BRCA1, treated with Olaparib (5 μM, 24 h) or DMSO. The cells were fixed and stained with anti-pRPA (T21) and propidium-iodide (DNA content). (Left) The figure is a representative of three independent experiments. (Right) Bar chart shows the mean ± SEM of percentages of pRPA(T21) positive cells from three independent experiments (*n* = 3). Asterisks indicate *P*-values obtained by one-way ANOVA (*** *P*< 0.001, **** *P*< 0.0001). (**B**,left) Representative images of immunofluorescence experiment of Rad51 foci formation in HeLa wild-type and POLE4 KO cells upon treatment of Olaparib (10 μM, 48 h), Scale bar, 10 μm. (Right) Quantification of Rad51 foci count in the indicated cell lines upon the indicated treatment. The experiment is representative of three independent repetitions. Asterisks indicate *P*-values obtained by one-way ANOVA (ns, not significant, **** *P*< 0.0001). (**C**) Cell survival assay of HeLa wild-type and POLE4 KO cells transfected with the indicated siRNA and treated with the indicated concentration of Olaparib for 7 days, with the treatment being changed once, before calculating the relative survival normalized to the untreated samples of each genotype. Mean ± SEM (*n* = 3). The figure is derived from three independent experiments. Asterisks indicate *P*-values obtained by two-way ANOVA (**** *P*< 0.0001). (**D**) Cell survival assay of HeLa wild-type and POLE4 KO cells downregulated of either BRCA1, 53BP1 or both of them using siRNA transfection and treated with the indicated concentrations of Olaparib for 7 days, with the treatment being changed once. The curves are normalized to the untreated condition corresponding to each genotype. Mean ± SEM (*n* = 3). The figures are derived from three independent experiments. Asterisks indicate *P*-values obtained by two-way ANOVA (ns, not significant, ** *P*< 0.01, **** *P*< 0.0001). (**E**) In POLE4-proficient cells, PARPi is synthetic lethal with BRCA1 deficiency, which is reversed upon loss of both BRCA1 and 53BP1 leading to PARPi acquired resistance. In POLE4-deficient background, the cells become sensitive to PARPi, and this sensitivity is further enhanced upon loss of BRCA1. Importantly, the acquired resistance to PARPi due to co-depletion of BRCA1 and 53BP1 can be bypassed in POLE4-deficeint cells, highlighting a potential therapeutic exploitation in the clinic. Created with BioRender.com

Since PARPi sensitivity was first described in BRCA-deficient cells displaying impaired HR (1,2), we aimed to check whether PARPi-induced replication stress response could be detected when BRCA1 was missing. Similar to POLE4 KO, downregulating BRCA1 resulted in increased levels of pRPA upon Olaparib treatment (Figure 6A). Strikingly, co-depletion of POLE4 and BRCA1 had a synthetic impact on pRPA levels compared to single depletion (Figure 6A). This suggests that POLE4 might function parallel to BRCA1, and that it is not part of the canonical HR pathway.

To confirm this hypothesis, we examined PARPi-induced Rad51 foci formation by confocal microscopy. The recombinase Rad51 is a crucial protein in the process of HR: following DNA end-resection, Rad51 binds ssDNA overhangs and leads the homology search and strand invasion to facilitate homology-directed repair (1,2). Consistent with previous reports describing impairment in HR, BRCA1-deficient cells displayed reduced Rad51 foci formation compared to the BRCA1 proficient controls (Supplementary Figure S6A, B). Conversely, POLE4 KO cells were able to efficiently form Rad51 foci upon Olaparib treatment, even to a higher extent than their wild-type counterpart (Figure 6B). This observation can be attributed to the elevation of ssDNA gaps we described previously in POLE4 KO following PARPi (Figure 3C, D).

Since POLE4 is not redundant in function with BRCA1, we reasoned that PARPi sensitivity could be potentiated if both proteins were missing. To that end, we downregulated BRCA1 in wild-type and POLE4 KO and challenged the cells with a low dose of Olaparib. BRCA1 depletion in POLE4 KO cells resulted in massive killing of these cells in comparison to the loss of either POLE4 or BRCA1 alone (Figure 6C), indicating that POLE4 might serve as a potential target for enhancing sensitivity of BRCA1-deficient tumors to PARPi.

A common mechanism for BRCA1-deficient tumors acquiring resistance to PARPi is the rewiring of HR through loss of the NHEJ factor 53BP1 (68–70). Given that sensitivity of POLE4 KO to PARPi is not going through defective HR, we sought to investigate whether targeting POLE4 could overcome PARPi resistance observed upon loss of 53BP1 in BRCA1-deficient cells (68–70). To that end, we utilized RNAi-mediated downregulation of either BRCA1, 53BP1 or their combination in wild-type and POLE4 KO cells. Consistent with previous reports, downregulating BRCA1 in wild-type cells sensitized them to Olaparib, which was rescued with combined depletion of BRCA1/53BP1 (Figure 6D, Supplementary Figure S6C). As mentioned earlier, BRCA1 depletion in POLE4 KO cells resulted in severe sensitization to Olaparib in comparison to missing either POLE4 or BRCA1 alone (Figure 6D). Significantly, the co-depletion of BRCA1/53BP1 did not rescue PARPi sensitivity of POLE4 KO as in the case of wild-type cells (Figure 6D, Supplementary Figure S6C), indicating that targeting POLE4 not only enhanced PARPi synthetic lethality in BRCA1-depleted cells but also bypassed the synthetic viability induced by reactivation of HR upon 53BP1 loss in BRCA1-compromised cells (69).

## Discussion

Our findings confirm that the deficiency of POLE4 results in heightened sensitivity to PARPi. While Olaparib inhibits both PARP1 and PARP2 (7), our data supports the notion that the toxicity of PARPi in POLE4 KO is primarily associated with PARP1 rather than PARP2, justifying the rationale behind the development of PARP1-specific inhibitors (71,72). The observed synthetic lethality between POLE4 deficiency and PARP inhibition is ascribed to the prolonged presence of inhibited PARP1 on chromatin rather than the blocked ADP-ribosylation activity of PARP1. This is substantiated by the fact that the depletion of PARP1 mitigates the toxicity of PARPi in POLE4 KO.

Based on our current understanding, the process of PARP1 trapping necessitates the presence of lesions that PARP1 can bind to, leading to its activation in the absence of PARPi. Considering the observed synthetic lethality, two scenarios can be hypothesized. On one hand, the absence of POLE4 might contribute to an increase in lesions that activate PARP1. Consequently, a higher prevalence of these activating lesions could result in more PARP1 trapping and increased sensitivity in the presence of PARPi. Here, one would anticipate elevated ADP-ribosylation levels. However, our findings reveal no increase either in S-phase PAR levels or in ADPr in response to DNA damage in POLE4 KO as compared to wild-type, therefore, they are more consistent with the alternative scenario suggesting that POLE4 plays a role in mitigating the toxicity induced by PARP trapping.

PARPi cytotoxicity is associated with the accumulation of ssDNA gaps (17,18). Such ssDNA gaps can arise from various sources, such as defective Okazaki fragments processing (17,52), or stalled replication forks (18). Unligated Okazaki fragments have been proposed to be a major source of PARP activity in S-phase. If POLE4 functions to ensure timely processing of Okazaki fragments, then its loss is expected to cause accumulation of ssDNA fragments even without PARPi treatment, such accumulation will be translated into increased PAR levels in S-phase cells just as in BRCA-deficient cells (17). However, POLE4 KO cells do not show increased S-phase PAR signal compared to their wild-type counterparts upon treatment with either PARGi or the combination of PARGi and Fen1i, the latter interfering with Okazaki fragment processing indicating that the loss of POLE4 does not increase the formation of Okazaki fragments.

Yeast studies identify a role of the POLϵ complex in the activation of S-phase checkpoint either through the C-terminal of the catalytic subunit or the accessory subunit Dpb4 in response to replication stress (25,73,74). Importantly, these accessory subunits also contribute to normal replication fork progression (24). Moreover, several reports have shown that the loss of POLE4 causes reduced replication origin activation in mice and worms (27,75,76). These replication defects remain, however, relatively mild unless these cells are subjected to replication stress inducers (27). These findings, together with our data that POLE4 KO cells elicit reduced replication rate under normal conditions and hypersensitivity to replication stress, as well as other previous reports (21,54,55), all point towards a key role of POLE4 in replication stress tolerance.

PARPi has been reported to increase fork speed, which induces replication stress (51). Further, PARP1 plays a role in the resolution of R-loops (58–60), whose function when inhibited may also interfere with efficient replication, a phenotype that was reported recently and correlated with PARPi sensitivity (56). Considering the reduced fork speed in POLE4 KO upon PARPi, it is tempting to speculate that the instability of fork progression due to POLE4 loss is potentiated when PARP1 is trapped on chromatin and interferes with efficient replication, which ultimately lead to the replication stress phenotype in POLE4 deficient cells. Reduced replication speed due to discontinuities of DNA synthesis requires efficient post-replicative repair and resolving of R-loops to alleviate the genomic stress accompanied by elevation of such DNA gaps (57,77). Our results suggest that impairment of these processes is likely to contribute to the low replication stress tolerance of POLE4 KO.

The arising ssDNA gaps in POLE4 KO are recognized by ATR and lead to its activation, the critical kinase in the protection against replication stress (78). ATR blocks cells from entering mitosis with unrepaired damage and reduces the replication rate to prevent potentiating replication stress (79). Along with this, several studies have shown that combining PARPi and ATRi synergistically kills BRCA 1/2 deficient cells by causing premature mitotic release (66,80). The toxicity of drug combinations could be further reduced through identifying novel genetic alterations that enhance susceptibility towards these drugs (54). Based on our results, POLE4 can serve as a target to enhance the sensitivity of cancer cells to the combination of PARPi and ATRi.

PARPi were reported to cause ATM activation (81,82). Our data further validate ATM activation as part of the canonical response to both PARPi and ATRi as it is evident in wild-type cells, and much increased in POLE4 KOs due to their increased sensitivity. Conversely, DNA-PK signaling remains inactive in wild-type cells following PARPi or ATRi treatment, while being excessively activated in POLE4 KO cells, contributing to some of the observed toxicity. Similar upregulation of DNA-PK signaling has been documented in HR-deficient cells exposed to PARPi (83).

Importantly, our results place POLE4 in a BRCA1-independent pathway underlying PARPi resistance. In contrast to BRCA1-deficient cells, sensitivity of POLE4 KO cells to PARPi is not rescued by the restoration of HR upon 53BP1 depletion (Figure 6E). Sensitivity of BRCA1-deficient tumors to PARPi can be attributed to three main mechanisms: (i) HR deficiency, (ii) loss of replication fork protection, (iii) defects in Okazaki fragments processing. POLE4 KO cells differ from BRCA1-deficiency in all these mechanisms. Genetic deletions of POLE4 have been identified in cases of malignant mesothelioma (84) and non–small cell lung cancer (85). Therefore, our data suggest that POLE4 might serve as a biomarker for identifying tumors that can respond to PARPi treatment regardless of their HR status.

## Supplementary Material

gkae439_Supplemental_File

## Data Availability

The data underlying this article are available in the article and in its online supplementary material. Further data underlying this article will be shared on reasonable request to the corresponding author.

## Notes

Present address: Rebecca Smith, Sir William Dunn School of Pathology, University of Oxford, Oxford OX1 3RE, UK.
