# Supplementary material for: The loss of DNA polymerase epsilon accessory subunits POLE3–POLE4 leads to BRCA1-independent PARP inhibitor sensitivity
Source: Nucleic Acids Res. 2024 Jun 3;52(12):6994–7011. doi: 10.1093/nar/gkae439 (PMC11229324; doi:10.1093/nar/gkae439)
Supplement: gkae439_Supplemental_File [file gkae439_supplemental_file.pdf]

Supplementary Materials

The loss of DNA polymerase epsilon accessory subunits POLE3-POLE4 leads to BRCA1-independent PARP inhibitor sensitivity

Hasan Mamar[1,2,$], Roberta Fajka-Boja[1,3,$], Mónika Mórocz[4], Eva Pinto Jurado[1,5,6], Siham Zentout[6], Alexandra Mihuț[1,5], Anna Georgina Kopasz[1,5], Mihály Mérey[1,5], Rebecca Smith[6,#], Abhishek Bharadwaj Sharma[7], Nicholas D. Lakin[7], Andrew James Bowman[8], Lajos Haracska[4], Sébastien Huet[6], Gyula Timinszky[1,*]

Supplementary Figures and their legends S1 – S6

Supplementary tables and their legends S1- S3

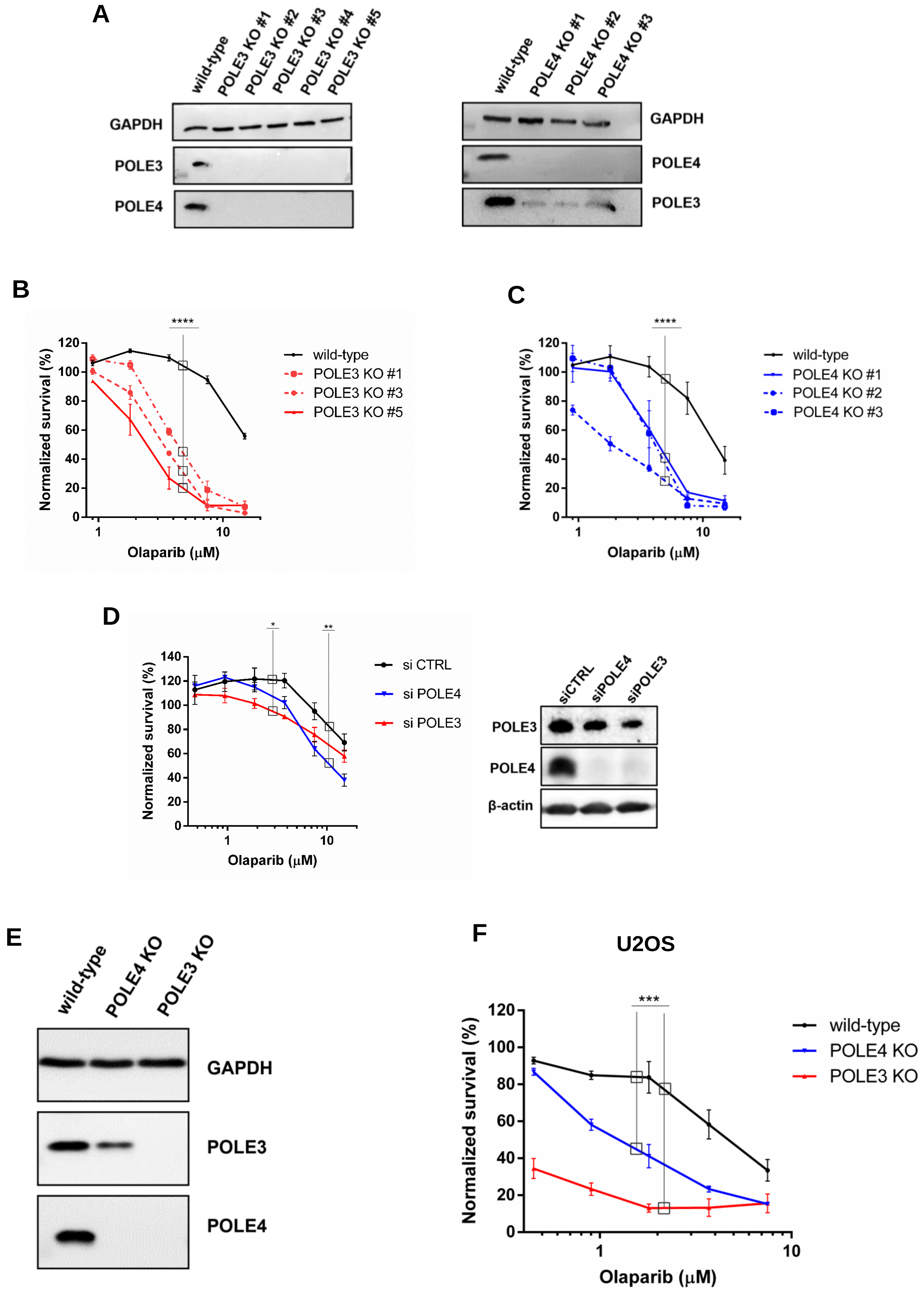

Supplementary Figure 1

Mamar et al.

Supplementary Figure 1:

(A) Western blot of different independent clones of (left) POLE3 KO and (right) POLE4 KO and their parental HeLa wild-type showing their POLE3 and POLE4 protein expression. GAPDH is used as a loading control.

(B, C) Cell survival assays demonstrating sensitivity of (B) POLE3 KO clones and (C) POLE4 KO clones to Olaparib treatment compared to their parental HeLa wild-type. The curves are normalized to the untreated condition corresponding to each genotype. PARPi treatment was refreshed once during the 7-day long experiment. Mean ± SEM (n=3). The figures are derived from three independent experiments. Asterisks indicate *p*-values obtained by two-way ANOVA (**** $p< 0.0001$).

(D) (Left) Cell survival assay showing Olaparib sensitivity of HeLa wild-type upon downregulation of POLE3 or POLE4 using siRNA transfection. PARPi treatment was refreshed once during the 7-day long experiment. The graphs show the relative survival normalized to the untreated samples of each transfection. Data are mean ± SEM (n=3) of triplicate samples from one representative out of three independent experiments. Asterisks indicate *p*-values obtained by two-way ANOVA (* $p< 0.05$, ** $p< 0.01$). (Right) Verification of POLE3 and POLE4 protein expression 72h post-transfection by Western blotting, using specific antibodies and GAPDH as loading control.

(E) Western blot of POLE3 and POLE4 knockouts generated in the U2OS cell line showing the importance of the accessory subunits for their stability. GAPDH is used as a loading control.

(F) Cell survival assays of U2OS wild-type, POLE3 and POLE4 knockout cells. The curves are normalized to the untreated condition corresponding to each genotype. The treatment lasted for 7 days and was refreshed once. Mean ± SEM (n=3). The figure is derived from three independent experiments. Asterisks indicate *p*-values obtained by two-way ANOVA (*** $p< 0.001$).

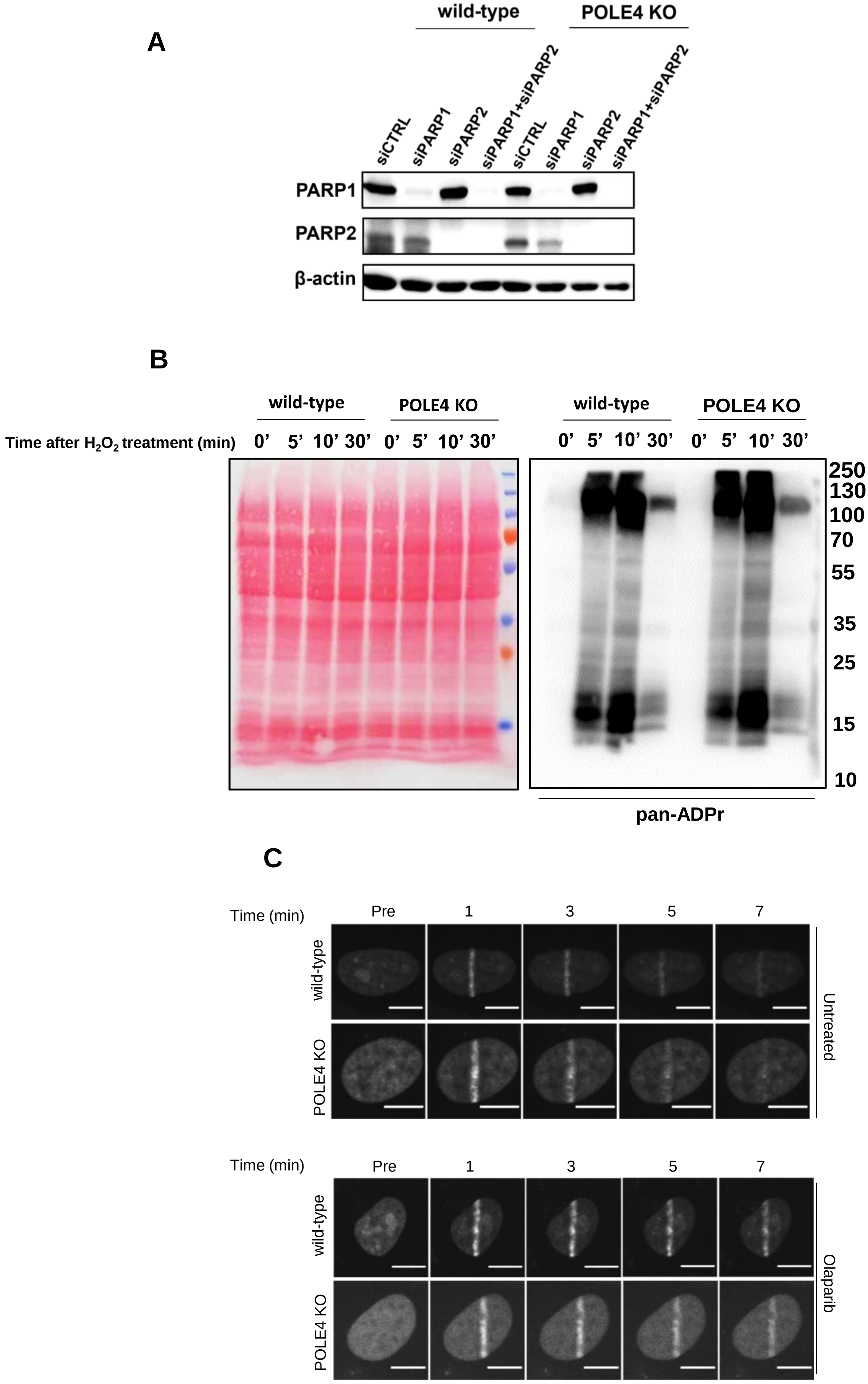
A
wild-type
POLE4 KO
siCTRL
siPARP1
siPARP2
siPARP1+siPARP2
PARP1
PARP2
β-actin
B
Time after H_2O_2 treatment (min)
0' 5' 10' 30'
250
130
100
70
55
35
25
15
10
pan-ADPr
C
Time (min)
Pre
1
3
5
7
Untreated
Olaparib

Supplementary Figure 2:

(A) Western blot of HeLa wild-type and POLE4 KO cells showing downregulation of PARP1, PARP2, or both due to transfection with the indicated siRNA. The cells were harvested for Western blot 48h post transfection. β-actin is used as a loading control.

(B) ADPr levels in both HeLa wild-type and POLE4 KO shown by Western blotting. The cells were treated or not with H_2O_2 (2 mM) for the indicated time points. ADPr signal was probed using pan-ADPr reagent (MABE1016). Ponceau staining is used as a loading control.

(C) Representative images of GFP-tagged PARP1 chromobody accumulation at sites of laser-induced damage in HeLa wild-type and POLE4 KO cells untreated (Top) or treated with Olaparib (30 nM) (Bottom). Scale bar, 10 μm.

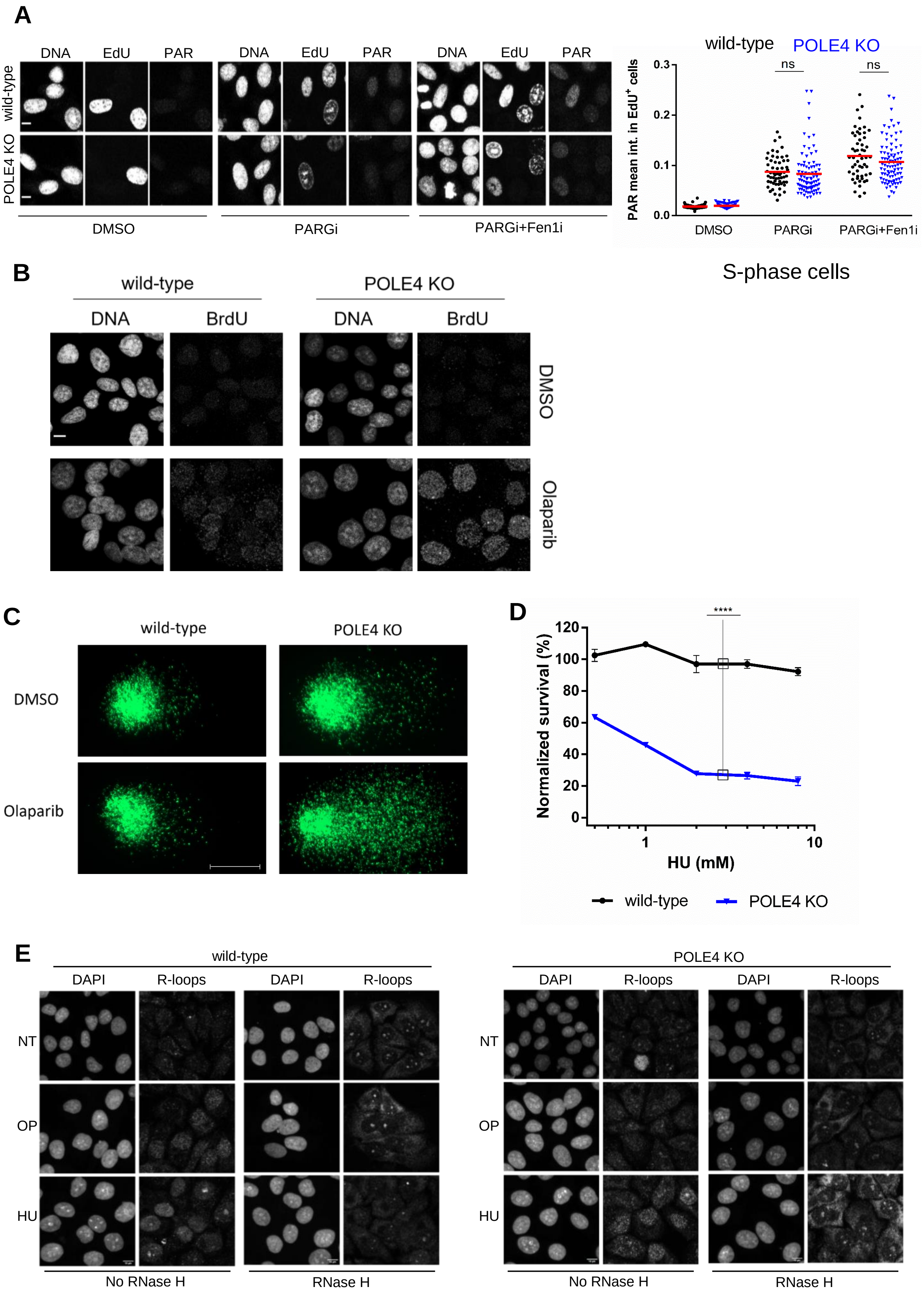

Supplementary Figure 3 **Mamar et al.**

Supplementary Figure 3:

(A) PAR signal in S-phase cells. HeLa wild-type and POLE4 KO cells were treated or not with PARGi (10 µM) or PARGi (10 µM) + Fen1i (10 µM) for 1h, and with EdU (10 µM, last 20 min) before fixation. PAR signal was detected using PAR-binding reagent (MABE1031). EdU click-it reaction was used to identify cells in S-phase. (Left) Representative images showing PAR signal in EdU-positive cells (indicative of S-phase). Scale bar, 10 µm. (Right) Quantification showing no significant difference in the mean intensity of PAR signal in S-phase cells between the two compared genotypes. The graphs are derived from a representative experiment out of three independent repetitions. Statistical analysis is done using one-way ANOVA (ns. Not significant).

(B) Representative images of immunofluorescence experiment of native BrdU staining. Cells with the indicated genotypes were incubated with BrdU (20 µM, 48h), then treated with Olaparib (10 µM, 24h) or the control vehicle DMSO. Scale bar, 10 µm.

(C) Representative images of BrdU comet experiment showing tail formation in POLE4 KO compared to HeLa wild-type upon treatment of Olaparib (20 µM, 24h), Scale bar, 20 µm.

(D) Cell survival assay showing POLE4 KO sensitivity to hydroxyurea (HU) treatment compared to HeLa wild-type. The curves are normalized to the untreated condition corresponding to each genotype. HU treatment was for 24h, then the cells were left to recover for 7 days in culturing medium. Mean ± SEM (n=3). The figure is derived from three independent experiments. Asterisks indicate *p*-values obtained by two-way ANOVA (**** $p < 0.0001$).

(E) Representative images of R-loop detection in untreated, Olaparib- (10 µM, 24h) or Hydroxyurea-treated (HU, 2 mM, 24h) wild-type or POLE4 KO HeLa cells, with or without RNase H treatment. Scale bar, 10 µM.

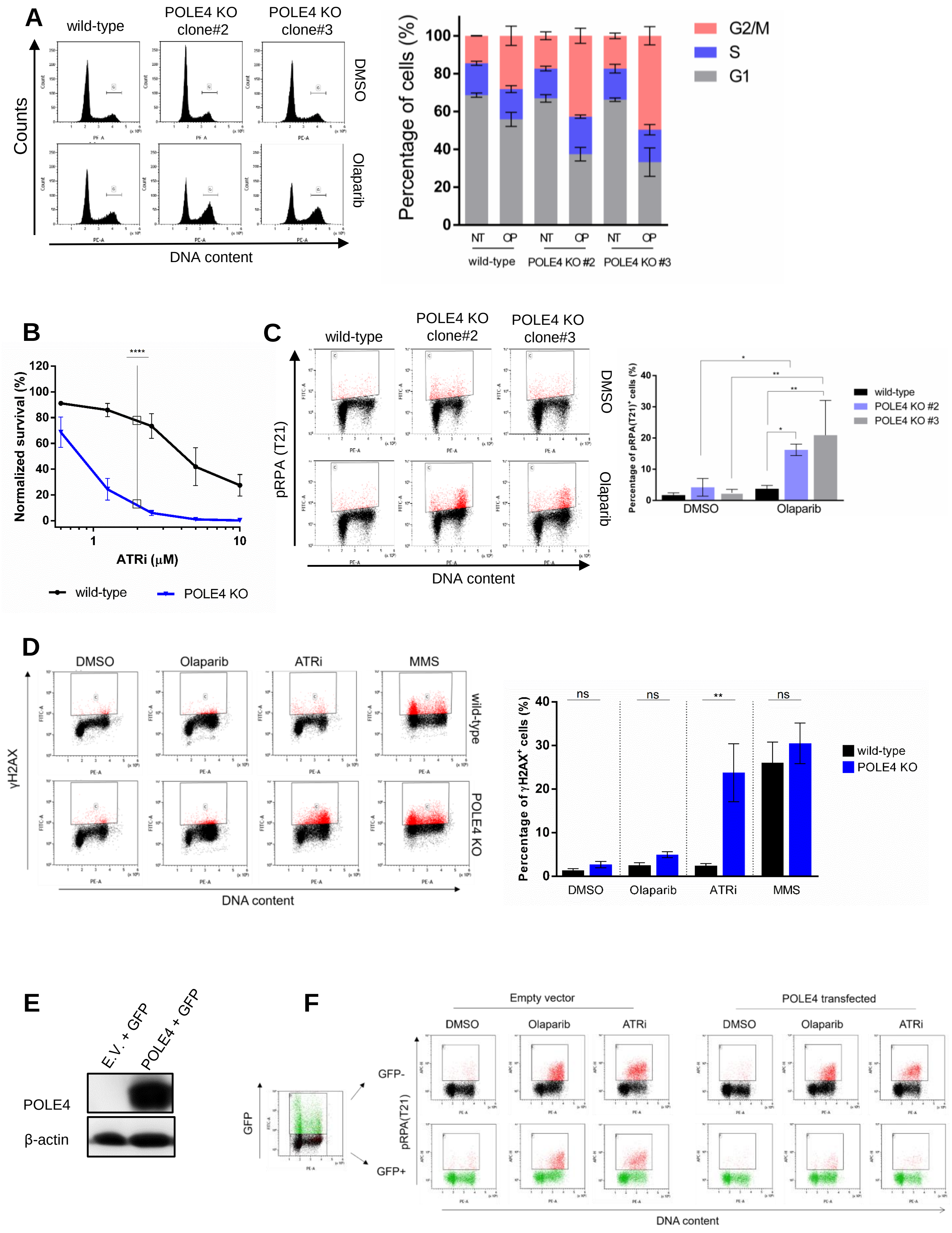

Supplementary Figure 4

Mamar et al.

Supplementary Figure 4:

(A) (Left) Representative FACS experiment showing cell-cycle profile of HeLa wild-type and different POLE4 KO clones with or without Olaparib treatment (5 μM, 24 h). (Right) Percentages of cells in G1, S or G2/M cell cycle phases are presented as mean ± SD of three independent experiments.

(B) Cell survival assays demonstrating enhanced sensitivity of POLE4 KO cells to ATRi treatment compared to their parental HeLa wild-type. The curves are normalized to the untreated condition corresponding to each genotype. ATRi treatment was refreshed once during the 7-day long experiment. Mean ± SEM (n=3). The figure is derived from three independent experiments. Asterisks indicate *p*-values obtained by two-way ANOVA (**** $p< 0.0001$).

(C) (Left) Representative FACS experiment showing RPA(T21) phosphorylation in HeLa wild-type and its different POLE4 KO clones with or without Olaparib treatment (5 μM, 24 h). (Right) Bar chart shows the mean ± SD of percentages of pRPA(T21) positive cells from three independent experiments, (n=3). Asterisks indicate *p*-values obtained by one-way ANOVA (* $p< 0.05$, ** $p< 0.01$).

(D) Flow cytometry showing γH2A.X levels of HeLa wild-type or POLE4 KO cells after culturing for 24h with Olaparib (5 μM), ATRi (5 μM) or 1h with MMS (0.1%). The cells were fixed and stained with anti-γH2A.X and propidium-iodide (DNA content). (Left) FACS images of a representative experiment are shown. (Right) Bar chart shows the mean ± SD of percentages of γH2A.X positive cells from three independent experiments, (n=3). Asterisks indicate *p*-values obtained by one-way ANOVA (ns: not significant, ** $p< 0.01$).

(E) Western blotting for verification of POLE4 protein expression 48h post-transfection with GFP and empty (E.V.) or POLE4 coding plasmids. β-actin was used as loading control.

(F) Detection of RPA(T21) phosphorylation in POLE4 KO cells after transient POLE4 expression. POLE4 KO cells were transfected with GFP and either empty (E.V.) or POLE4 coding plasmids for 48h, then treated with Olaparib (5 μM), ATRi (5 μM) or with DMSO for 16h. The cells were fixed and stained with anti-pRPA(T21) and propidium-iodide (DNA content). RPA(T21) phosphorylation is shown in cells gated to GFP positive and negative populations during flow cytometry analysis. FACS images of a representative of three independent experiments are shown.

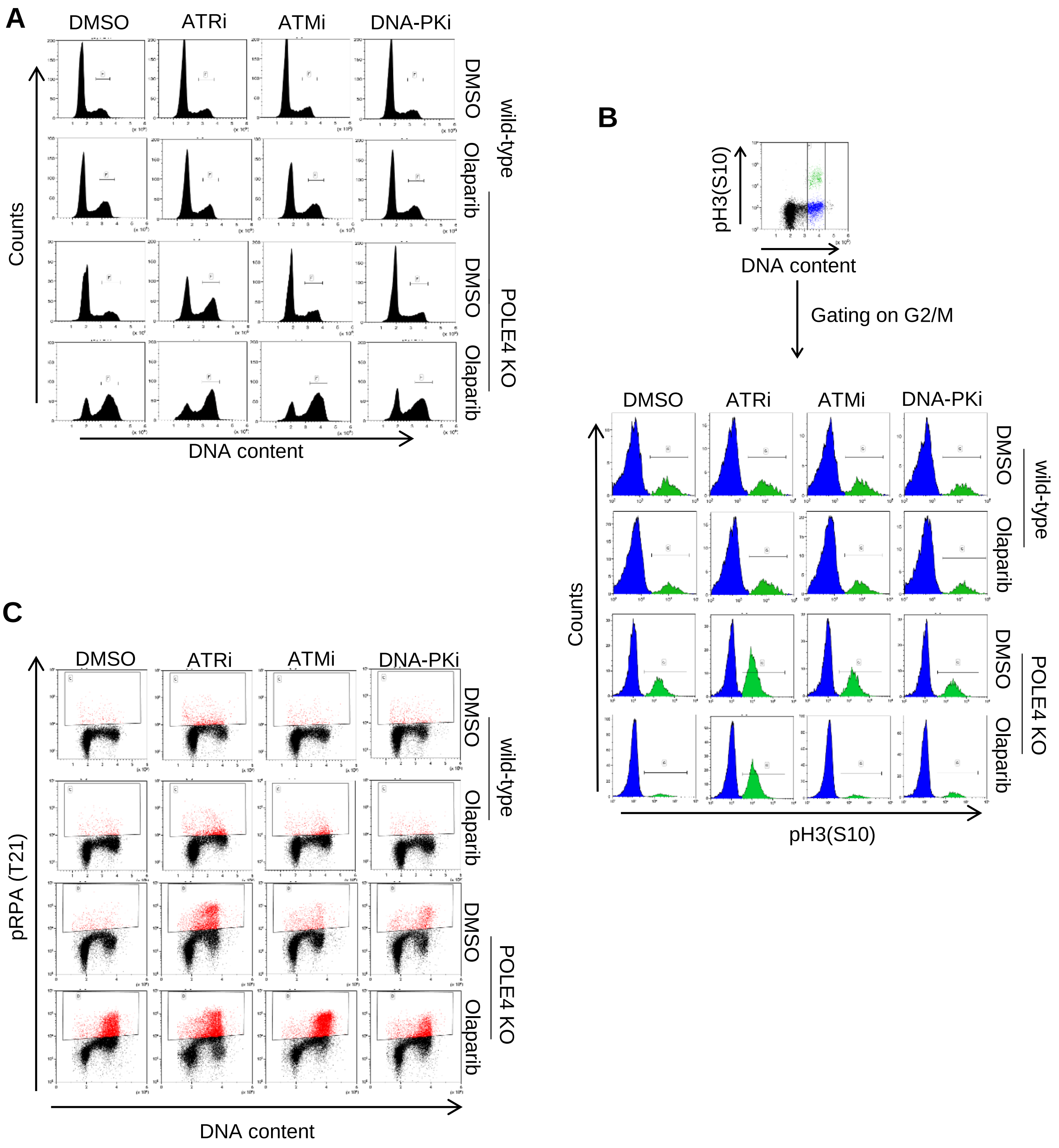

Supplementary Figure 5

Supplementary Figure 5:

(A) Representative flow cytometry experiment showing cell-cycle profile of HeLa wild-type and POLE4 KO cells after 24h treatment with Olaparib (5 µM) and/or ATRi (5 µM), ATMi (5 µM), DNA-PKi (5 µM). DMSO was used as a solvent control. The cells were fixed and stained with propidium-iodide (DNA content). The figure is a representative of five independent experiments.

(B) Representative FACS experiment for distinguishing the mitotic cells by positive staining of pH3(S10) (green) from G2 phase cells (blue). HeLa wild-type or POLE4 KO cells were treated or not with Olaparib and/or ATRi, ATMi, DNA-PKi (5 µM, 24 h), and then stained with anti-pH3(S10) and propidium-iodide. (Top) Gating on G2/M was conducted based on DNA content. (Bottom) Histograms show the pH3(S10) fluorescent intensity of cells in G2/M gate. DMSO was used as a solvent control.

(C) Flow cytometry of HeLa wild-type and POLE4 KO cells after 24h treatment of Olaparib (5 µM) and/or DNA-PKi (5 µM), ATRi (5 µM) or both. DMSO was used as a solvent control. The cells were fixed and stained with anti-pRPA (T21) and propidium-iodide (DNA content). The figure is a representative of four independent experiments.

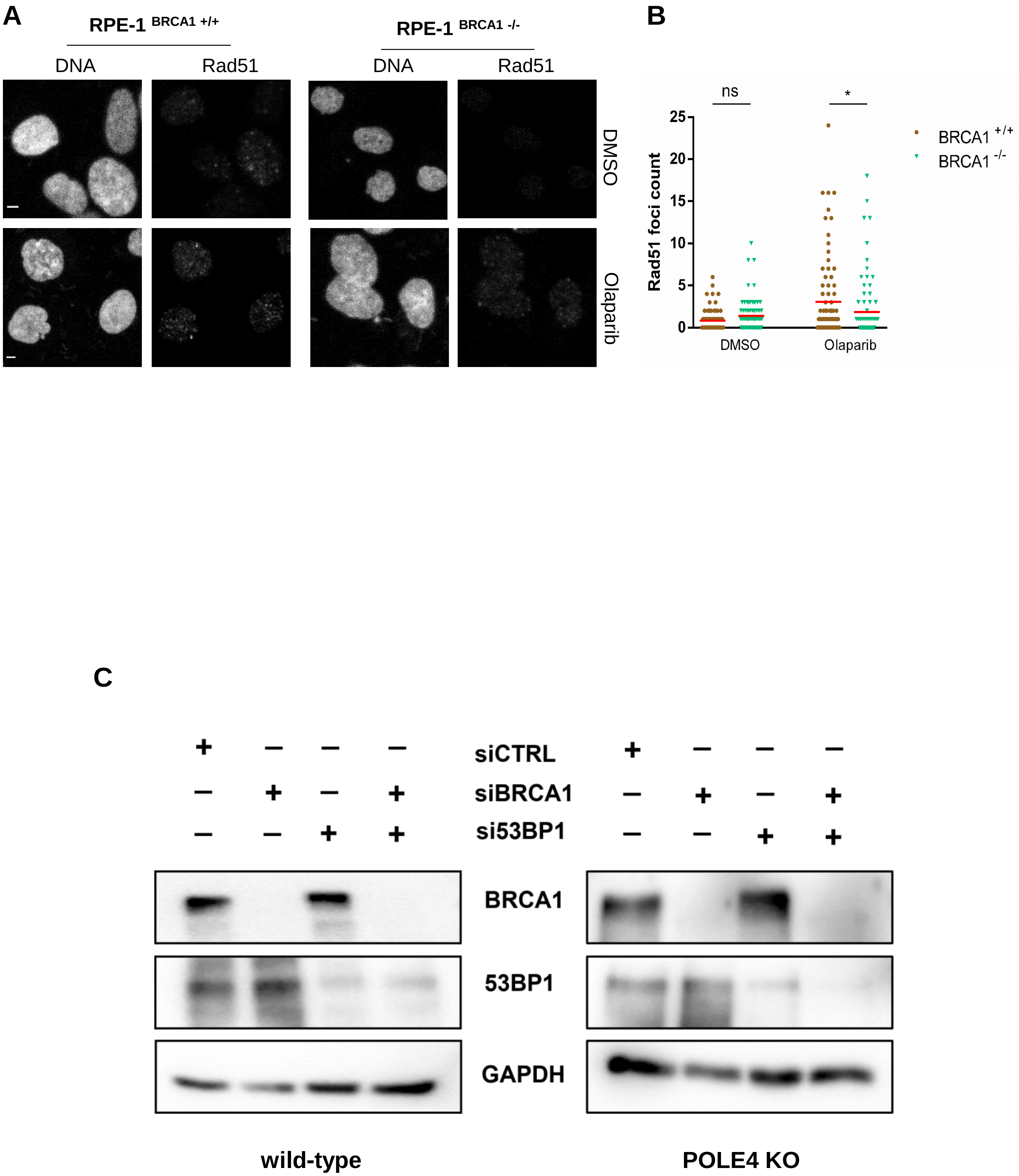
A
RPE-1 BRCA1 +/+
DNA
Rad51
RPE-1 BRCA1 -/-
DNA
Rad51
DMSO
Olaparib
B
ns
*
Rad51 foci count
25
20
15
10
5
0
DMSO
Olaparib
BRCA1 +/+
BRCA1 -/-
C
siCTRL
siBRCA1
si53BP1
BRCA1
53BP1
GAPDH
wild-type
POLE4 KO

Supplementary Figure 6:

(A) Immunofluorescence experiment of Rad51 foci formation in RPE-1 BRCA1-deficient cells and their wild-type upon treatment with Olaparib (10 μM, 48h), Scale bar, 10 μm.

(B) Quantification of Rad51 foci count in RPE-1 BRCA1-deficient cells and their wild-type upon treatment with Olaparib (10 μM, 48h). The experiment is representative of three independent repetitions. Asterisks indicate *p*-values obtained by one-way ANOVA (ns. Not significant, * $p < 0.05$).

(C) Representative Western blot of HeLa wild-type and POLE4 KO cells demonstrating depletion of the indicated proteins 48h post treatment with the indicated siRNA. GAPDH is used as a loading control.

Supplementary tables and their legends

Table S1. Dharmacon smart pool siRNA

Target	Name	Reference
CTRL	ON-TARGET plus Non-targeting Control siRNAs	D-001810-01-20
PARP1	ON-TARGET plus Human PARP1 siRNA	L-006656-03-0005
PARP2	ON-TARGET plus Human PARP2 siRNA	L-010127-02-0005
BRCA1	ON-TARGET plus Human BRCA1 siRNA	L-003461-00-0005
53BP1	ON-TARGET plus Human TP53BP1 siRNA	L-003548-00-0005
POLE4	ON-TARGET plus Human POLE4 siRNA	L-009850-02-0005
POLE3	ON-TARGET plus Human POLE3 siRNA	L-008460-01-0005

Table S2. Antibodies and antibody-like reagents used in this study

Target	Host	Company	Reference	Dilution in IF	Dilution in FACS	Dilution in WB
53BP1	Rabbit	Abcam	ab36823	-	-	1:3000
ADP-ribose (Pan reagent)	Rabbit	Millipore	MABE1016	-	-	1:1000
Anti-DNA-RNA Hybrid Antibody, clone S9.6	Mouse	Sigma-Aldrich	MABE1095	1:100	-	-
Beta-Actin	Mouse	Thermo Fisher Scientific	MA5-15739	-	-	1:5000
BrdU	Mouse	Santacruz	sc-32323	1:200	1:200	-
BrdU	Mouse	Becton Dickinson	347580	1:400	-	-
BrdU	Rat	Abcam	6326	1:400	-	-
CHRAC15	Rabbit	ABclonal	A14896	-	-	1:1000

gamma H2A.X (phospho S139)	Rabbit	Abcam	ab81299	-	1:250	-
GAPDH	Rabbit	Thermo Fisher	PA1-16777	-	-	1:4000
PARP1	Rabbit	Abcam	ab32138	-	-	1:2000
PARP2	Rabbit	Proteintech	55149-1-AP	-	-	1:2000
pATM	Rabbit	Abcam	ab81292	-	-	1:5000
pATR (T1989)	Rabbit	Abcam	ab223258	-	-	1:1000
pDNAPK	Rabbit	Invitrogen	PA5-78130	-	-	1:1000
p-Histone H3(Ser10)	Mouse	Invitrogen	MA5-15220	-	1:200	-
POLE3	Rabbit	ABclonal	A6469	-	-	1:1000
POLE4	Rabbit	ABclonal	A9882	-	-	1:1000
Poly(ADP-ribose) WWE-domain	Rabbit	Millipore	MABE1031	1:200	-	-
pRPA (S33)	Rabbit	Fortis Life sciences	A300-249A	-	-	1:1000
pRPA (S4/8)	Rabbit	Fortis Life sciences	A300-245A	-	-	1:1000
pRPA (T21)	Rabbit	Abcam	ab109394	-	1:2000	-
Rad51	Rabbit	Abcam	ab133534	1:1000	-	-
Secondary antibodies						
Alexa Fluor 488 anti-rabbit IgG	Goat	Invitrogen	A11008	1:500	1:500	-

Alexa Fluor 488 anti-mouse IgG	Goat	Invitrogen	A11001	1:500	1:500	-
Alexa Fluor 488 anti-rat IgG	Goat	Biotium	200023	1:500	-	-
Alexa Fluor 555 anti-rabbit IgG	Goat	Invitrogen	A21428	1:500	1:500	-
Alexa Fluor 555 anti-mouse IgG	Goat	Invitrogen	A21422	1:500	1:500	-
Alexa-Fluor-546 anti-mouse IgG	Goat	Thermo Fisher	A21123	1:500	-	-
Alexa Fluor 647 anti-mouse IgG	Goat	Invitrogen	A21235	-	1:500	-
Anti-Mouse IgG-HRP	Goat	Invitrogen	A16066	-	-	1:5000
Anti-Rabbit IgG-HRP	Goat	Thermo Fisher	G-21234	-	-	1:5000

Table S3. Inhibitors used in this study

Inhibitor	Commercial name	Company	Reference
AG014699	Rucaparib	MedChem Express	HY-10617A
AZD2281	Olaparib	Selleck Chemicals	S1060
BMN-673	Talazoparib	MedChem Express	HY-16106
KU-55933	ATMi	Selleck Chemicals	S1092
LNT1	Fen1i	Tocris	6510
NU7441	DNAPKi	Selleck Chemicals	S2638
PDD 00017273	PARGi	MedChem Express	HY-108360
VE-821	ATRi	Selleck Chemicals	S8007
